# Life-cycle health effects of compulsory schooling

**DOI:** 10.1007/s10198-025-01884-2

**Published:** 2026-01-12

**Authors:** Johannes Hollenbach, Hendrik Schmitz, Beatrice Baaba Tawiah

**Affiliations:** 1 RWI - Leibniz Institute for Economic Research, Hohenzollernstr. 1-3, 45131 Essen, Germany; 2 Department of Economics, Paderborn University, Paderborn, Germany; 3 Munich Research Institute for the Economics of Aging and SHARE Analyses, Leopoldstraße 139, 80804 München, Germany

**Keywords:** Education, Health, Life-cycle effects, Compulsory schooling, I10, I12, I21

## Abstract

We study the effect of education on health (hospital stays, number of diagnosed conditions, poor or bad self-rated health, and body mass index) over the life cycle, using German compulsory schooling reforms as a source of exogenous variation. Our results show clear correlations between educational attainment and better health across all age groups (30 to 74). However, we do not find causal relationships between additional schooling and health or health care utilization, neither earlier nor later in life. A simulated ex-post power analysis shows that this is not due to a lack of statistical power. One reason for the absence of effects may be that the studied compulsory schooling reforms succeeded in raising the educational attainment of the target group - individuals at the lowest educational margin - but did not lead to healthier employment opportunities.

**Supplementary Information:**

The online version contains supplementary material available at 10.1007/s10198-025-01884-2.

## Introduction

Estimating the effects of education on socioeconomic outcomes has been an important part of applied microeconometrics over the past three decades. While most of the literature has focused on labor market outcomes, health effects have also been investigated. In Table 3 in the Appendix we list 24 studies that estimate health effects of education and use instrumental variable estimation methods for identification. More than half of these studies find no statistically significant effects overall or in relevant subgroups. All of these studies have in common that they aggregate effects across age groups, often over several decades. However, this may miss relevant patterns. Kaestner et al. [1] extend the classic [2] model of demand for health and conclude that “it is unlikely that the relationship between education and health is constant over the life cycle and that education is likely to have little effect on health at younger ages when there is little depreciation of the stock of health” [1]. Thus, an estimated small and insignificant effect averaged over younger and older individuals does not necessarily imply that health is not causally affected by education. It may well be that the effect occurs late in life and that the aggregate average is obscured by a zero effect for younger individuals.

It is well known that the socioeconomic status-health gradient increases over the life cycle [3, 4]. This descriptive pattern has also been shown more specifically for the education-health gradient. For example, Kaestner et al. [1] find no differences in mortality by education until the age of 60, after which hazard rates diverge by education. In contrast, they find an education-morbidity gradient only for the 45-60 age group, but explain this with possible selective mortality. Bijwaard et al. [5] find an increasing difference in mortality between those with primary education and those with more than primary education, mostly after the age of 60. They find that the differences are mainly due to selection effects (based on cognitive ability) at early ages, while the role of education increases after age 60. Leopold and Leopold [6] find differences in self-rated health between higher and lower educated individuals from ages 30 to 80, which increase from age 50 (for men). Ross and Mirowsky [7] find a gap in physical impairment between the well-educated and the poorly educated over the life cycle, which is more pronounced for women. These studies provide a descriptive picture of the education-health gradient over the life cycle, but do not claim causality.

We are aware of only two studies that explicitly look at causal health effects of education by age-group, thus allowing to take on a life-cycle perspective of health effects.^1^ Clark and Royer [10] find that the increase in minimum school leaving age from 14 to 15 in 1947 and from 15 to 16 in 1972 in Britain did not affect mortality overall, but also not within separate 5-year age groups between 20-24 and 65-69. Gehrsitz and Williams Jr [11] study the effects of the same 1972 reform but restrict the analysis to Scotland and report results by age for 30-55 year olds. They find no effect on self-reported health, but a reduction in hospital admissions for selected conditions. This is mainly true for men and starts after the age of 40.

We contribute to the literature by adding to that very small number of studies that uses exogenous variation to disentangle education effects on health by age. We use a different schooling reform in a different country compared to [10] and [11]. More importantly, even though [10] also study effects on health below levels of mortality (subjective health and health behavior), only for mortality they compute effects by age groups. In contrast, we show age-specific effects for health measures such as subjective health, diagnoses, body weight, and health-care utilization. Regarding [11], our main advancement is that we study effects up to age 74 while they study effects up to age 55.

In our study, the exogenous variation is derived from compulsory schooling reforms in West Germany. These reforms were introduced for birth cohorts between 1931 and 1954 and vary across federal states. We use data from the German Socio-Economic Panel (SOEP), an ongoing representative survey that has been conducted since 1984. Our dataset covers this entire 39-year period and allows us to follow individuals born around the pivotal reform cohorts over many decades and to estimate both short- and long-term effects of education on health within the same framework and dataset. We use instrumental variables-techniques to estimate effects for 5-year age groups between the age of 30 and 74. This enables us to identify when potential health returns to education begin to materialize or grow. Estimating life-cycle health effects using outcomes below the level of mortality would not be possible with any available administrative data set in Germany.

Our results show a clear positive correlation between health and education at all ages. An additional year of education is associated with a lower likelihood of having been hospitalized in the past year, a lower number of diagnosed illnesses, a lower likelihood of rating one’s health as poor or bad, and a lower BMI. The associations between education and number of illnesses and poor self-rated health appear to peak between the ages of 50 and 65. The education-health gap shows signs of increasing with age for hospital stays and BMI. However, when we examine the causal relationship, we find that education seems to have no effect on health or healthcare utilization. One exception may be self-rated health, where the effects increase throughout the life cycle such that the point estimates for the four oldest age groups may be economically relevant compared to the mean. Yet, they are statistically insignificant. The estimates for all other outcomes are very small and also not statistically significant. We show that our sample does not seem to suffer from power problems. Our simulated ex-post power analysis following [12] indicates that we could reliably detect true 2SLS effects of education on self-rated health, even if they were less than half the size of our estimated coefficients. Furthermore, we demonstrate that, even for the BMI analysis, for which we have considerably fewer observations per age group, we could theoretically detect very small effects, should they be present. Nevertheless, a limited sample size within each age group remains one of the limitations of this study.

To further corroborate why we do not see lasting health effects from the reform, we also investigate its impact on likely channels, namely income, employment, health behavior, as well as preferences and traits. We find that the reform increased the probability of being employed, but the effect on income is at best uncertain. The reform did not lead to healthier employment opportunities for compliers or to changes in alcohol or tobacco use, physical activity, or diet. It also did not affect risk-taking tendencies or locus of control.

One may argue that it comes at no surprise that the effects are modest at best. Previous literature has shown zero to very small effects of the German compulsory schooling reform on outcomes such as health, cognition, or income. In their review, Galama et al. [13] conclude that compulsory schooling reforms seem to affect health only if either there are effects on student’s peer groups, or if there are monetary returns to the reform. Both are not the case for the German schooling reform even though the debate whether there have been monetary returns or not is still ongoing [14]. Moreover, even though for a different measure of education, namely university education, Kamhöfer et al. [15] show how individuals with the lowest desire to take education do not seem benefit from education in terms of income or cognition. The effects we identify here are also for the individuals at the lowest margin of willingness to take education. Yet, we argue that this previous empirical evidence only addresses aggregate age effects and, ultimately, it is still an empirical question whether zero or moderate aggregate effects may mask larger health effects at higher age groups.

This paper is structured as follows. In the “Institutional framework and data” section we present the institutional framework, data, and descriptive statistics including OLS results. In the “Instrumental variables estimations by age group” section we show and discuss the main results: instrumental variables estimations for different age groups. Furthermore, we provide heterogeneous effects by gender, robustness checks, the power analysis, and inspect panel attrition. In the “Effects on non-health outcomes” section we explore possible reasons for the zero effects. We conclude in the “Conclusion” section.

## Institutional framework and data

### Institutional framework

In Germany, children enter primary school at the age of six. After four years in primary school they attend one of the three secondary school tracks. Secondary schools in Germany can generally be differentiated into basic (*Hauptschule*), intermediate (*Realschule*) and high schools (*Gymnasium*). The basic track (up to 8th or 9th grade) prepares students for apprenticeship, the intermediate track (up to 10th grade) qualifies students for apprenticeship or training in white-collar jobs, and the high school certificate (up to 12th or 13th) gives access to academic education in colleges or universities. Before the German educational reform that occurred in West Germany from 1946 to 1969, basic track schools covered grades five through eight, and about 75 percent of students attended these schools [16]. The reform increased the number of compulsory school years from eight to nine, which led basic track schools to add a ninth grade. Rather than stretching the material from grades 5-8 across four school years, students were introduced to additional material. The curriculum was adapted to resemble what was taught in the intermediate track [17]. Decisions and policies regarding the educational system in Germany are made at the federal state level. Nevertheless, the subjects taught to ninth-grade students did not vary across states and remained consistent with those taught in previous school years [16]. Additionally, a new subject, work studies (*Arbeitslehre*), was introduced in the basic track to better prepare students for the job market (e.g., through company visits or internships) and enable them to make more informed career choices [17]. No new degree was introduced, so the reform did not grant these students access to new types of jobs. However, the goal of more closely aligning the curricula of the basic and intermediary tracks until grade nine was to make it easier for students who originally started in the basic track to obtain an intermediary degree after grade 10 [17]. Nevertheless, switching between tracks was rare and most lateral movement between tracks took place by grade seven [18].

The reform was implemented at different times by the various states. Some states introduced the compulsory ninth grade earlier, while the majority only did so due to the Hamburg Accord of 1964, which sought to coordinate school calendars and set common rules, including compulsory attendance. See Table 1 for the reform years. The motivation for reforming the lower track shifted from an initial lack of labor market opportunities right after the Second World War to increasing education, skills, and maturity of students due to a growing need for more skilled graduates [16].

Several studies have already examined the impact of the reform on different domains. Since a majority of students attended the lower track, it had a large effect on years of schooling. For example, Kemptner et al. [19] find that the reform increased schooling by about 0.6–0.7 years. Kamhöfer and Schmitz [20] report an increase of 0.9–1 additional year of schooling. The evidence on employment effects is mixed. Pischke and Von Wachter [16] find no effects on employment probability, while [21] identify an increase in the probability of self-employment, but no effect on the probability of holding a blue- or white-collar job. The debate on wage effects is not yet settled. Pischke and Von Wachter [16] find zero effects with [20] confirming this finding using different data. However, a more recent study by [14] uncovers evidence of wage increases for compliers ranging from 6.7 to 11 percent, depending on the specifications and sample characteristics. In terms of health effects, Kemptner et al. [19] estimate a positive effect on self-rated health for men and a general negative effect on weight problems. In contrast, Begerow and Jürges [22] shows that the reform had no or very small impacts on a wide range of diagnosed conditions, using detailed claims data. The reform has also led to a decrease in fertility in Germany, as evidenced by [21]. This decline was primarily driven by a delay in first births, with no subsequent catch-up effects observed. Furthermore, there is evidence that the additional education from this reform improved sentiment towards immigration later on, but did not affect democratic values or political participation [23, 24].

### Data and sample

For our analysis we use the German Socioeconomic Panel (SOEP), which is a comprehensive representative longitudinal study of households in Germany. Established in 1984, it contains yearly information on around 30,000 respondents in nearly 15,000 households [25]. We use version 40 containing yearly information from 1984 to 2023 [26]. In addition to the outcomes we study, the data includes information on age, gender, the state in which an individual attended school, years of schooling and type of schooling completed. We use educational degree to infer years of schooling, as this is our explanatory variable of interest.

We restrict the sample to individuals born five years before and after the pivotal cohorts – that is, the first birth cohorts that were affected by the reform. Table 1 reports the reform years and shows how the age range of individuals we can theoretically identify effects for differ by federal states. For instance, for the outcome variables available from 1984 to 2023 in the SOEP, the youngest possible age is 25 for a person from Bavaria, born in 1959 (pivotal cohort + 5 years), observed in 1984. The oldest possible age is 97 for a person from Hamburg, born in 1926, observed in 2023. In our analysis below, we will form 5-year age groups to estimate effects. We restrict the sample to individuals between 30 (starting with age group 30-34) and 74 (for age group 70-74) years to make sure that effects for certain age groups at the higher and lower ends are not completely driven by individuals from single federal states.

**Table 1:** Reform years, corresponding first birth cohorts and ages

Federal State	Pivotal birth cohort	Reform year	Youngest age in 1984	Oldest age in 2023
Schleswig Holstein	April 1932	April 1947	47	96
Hamburg	April 1931	April 1946	48	97
Lower Saxony	April 1947	April 1962	32	81
Bremen	April 1944	April 1959	35	84
North Rhine-Westphalia	April 1951	April 1966	28	77
Hesse	April 1951	April 1966	28	77
Rhineland Palatinate	April 1952	April 1967	27	76
Baden-Württemberg	April 1952	April 1967	27	76
Bavaria	August 1954	August 1969	25	74
Saarland	April 1943	April 1958	36	85

Source: Reform years according to [22]. Youngest age in 1984 calculated as follows: 1984 - pivotal cohort - 5. Oldest age in 2023 calculated as follows: 2023 - pivotal cohort year + 5

In the Supplementary Materials, we extend the analysis to also include data from the Microcensus, the Survey of Health Ageing, and Retirement (SHARE), and the German National Educational Panel Study (NEPS). This increases the sample size but comes at the cost of pooling different data sources. Qualitatively, the results remain unaffected by this.

### Outcome variables and descriptive statistics

The health outcomes we consider are hospital stay in the previous year, number of illnesses diagnosed, poor self-rated health and the body mass index. More specifically, *Hospital stay* is an indicator variable based on the question whether a person was admitted at a hospital for at least one night the previous year. The number of illnesses diagnosed (called *diagnoses* from now on) is constructed from a question asking if an individual has ever been diagnosed by a doctor with one or more illnesses from a list of illnesses. The 13 illnesses asked are sleep disturbance, diabetes, asthma, heart disease, cancer, stroke, migraine, high blood pressure, depressive psychosis, dementia, joint disorder (also osteoarthritis, rheumatism), chronic back complaints and other illnesses. We count the number of diagnoses. *Poor or bad health* is based on the 5-point scale of current self-rated health status, that ranges from one (very good) to five (bad). Our outcome is an indicator variable equal one if an individual’s self-assessed health falls within the two worst categories. In the Appendix, we also present results for choosing the lowest category (*bad health*, Fig. 8). The final outcome is the body mass index (*BMI*), the ratio of a person’s weight (in kilograms) and squared height (in meters).

Table 2 presents descriptive statistics for all outcome variables. Because some outcomes are not measured in every wave, sample sizes differ across variables. Hospitalization has the largest sample (84,043 observations), while the number of diagnoses has the smallest (15,913 observations). On average, 12% of respondents spent at least one night in the hospital during the previous year, 20% report being in poor or bad health, and participants have 1.8 out of 13 possible conditions. The mean BMI in the sample is 26.75. Descriptive statistics for treatment status, instrument status, and additional characteristics are based on the hospitalization sample, as it is the most comprehensive and includes individuals aged 30 to 74. In this sample, respondents have an average of 10 years of schooling, are slightly more likely to be born in or after school reform years, have a mean age of 53.9 years, and are almost evenly split between men and women.

To get a better understanding of how the sample compares to the general population, we compare it to the full SOEP sample as well as external benchmarks featuring representative means in Table 4. That table also includes additional socioeconomic variables. Due to the limited availability of external benchmarks and to credibly compare data across multiple sources, we focus on one year: 2022. On average, the means for the two outcomes for which we have external benchmarks, hospitalization and BMI, are very close in all three data sources. The full SOEP sample has a slightly lower percentage of individuals who rate their health as poor or bad, as well as a lower number of chronic conditions. However, individuals in our sample are much older than those in the full SOEP sample or in external benchmarks. Consequently, they are far less likely to be employed in 2022, and more likely to be married. They are also more likely to have a lower educational degree. Nevertheless, their average years of schooling are fairly close to those of the full SOEP sample.

**Table 2:** Descriptive statistics

	Mean	SD	Min.	Max.	Observations
*Outcome variables*					
Hospitalization	0.12	0.33	0	1	84043
Poor or bad health	0.20	0.40	0	1	72299
Number of diagnoses	1.80	1.65	0	11	15913
BMI	26.75	4.57	13.70	62.86	27458
*Treatment and instrument*					
Years of schooling	9.98	1.68	8	13	84531
Reform	0.55	0.50	0	1	84531
*Other information*					
Age	53.94	10.94	30	74	84531
Female	0.51	0.50	0	1	84531

*Notes*: This table presents summary statistics. The outcome variables are defined as follows: Hospitalization is an indicator variable for whether a person was hospitalized for at least one night in the past year. Poor or bad self-rated health is a binary indicator of checking one of the two lowest of five possible categories of self-rated health. Number of diagnoses is a count variable of the total number of diagnosed (chronic) conditions and diseases from a list of 13 possible options. BMI is weight (in kilograms) divided by squared height (in meters). The statistics for age, gender, years of schooling, and reform are based on all observations with nonmissing information for at least one outcome variable

### OLS results

**Fig. 1:**
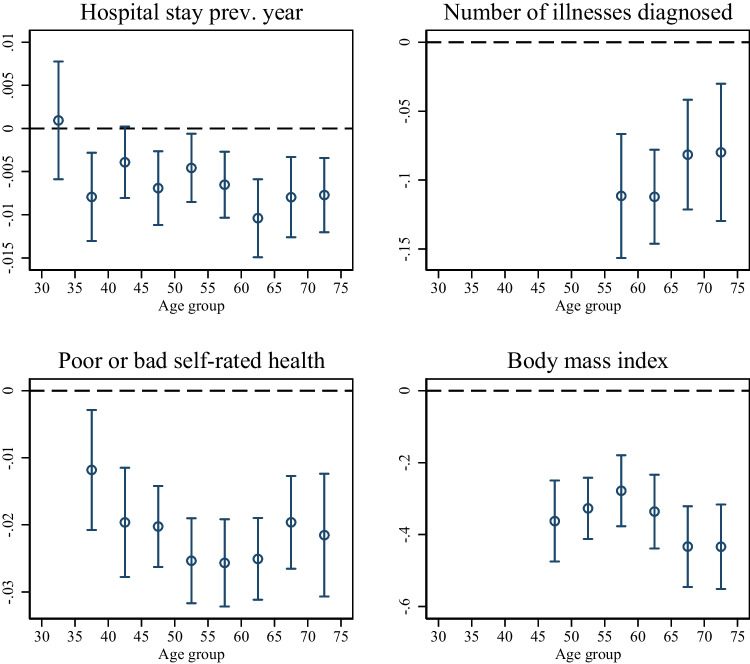
OLS results. *Notes:* This figure shows estimates of the association between years of schooling and four health outcomes across up to nine age groups. Hospital stay is an indicator variable for whether a person was hospitalized for at least one night in the past year. Number of diagnoses is a count of the total number of chronic conditions and diseases diagnosed from a list of thirteen possible options. Poor or bad self-rated health is an indicator variable for selecting one of the two lowest of the five possible self-rated health categories. BMI is weight (in kilograms) divided by height squared (in meters). Own calculations based on data from SOEP and our sample selection, as outlined in “Institutional framework and data” section. We visualize point estimates of the coefficients $\beta _g$ based on Eq. 1 with 95 % confidence intervals. Coefficients plotted between 30 and 35 represent age group 30-34, and so on. Some age groups are missing due to data constraints. Standard errors clustered at state*×*year of birth level

We estimate the association between education and health outcomes using an OLS regressions of the following form:

1 $$\begin{aligned} H_{ist}= & \sum _{g}{\beta _g Yed_{is} \times agegroup_{it}} + \beta X_{ist} + \varepsilon _{ist} \end{aligned}$$

where $H_{ist}$ is a health outcome of individual *i* who attended school in state *s*. $Yed_{is}$ is the number of years of schooling of an individual. To flexibly account for the correlation between schooling and health, we define 5-year age groups, denoted $agegroup_{it}$, as follows 30-34, 35-39, 40-45,*…*, 70-74.^2^ The vector *X* includes age dummies (in years) to flexibly control for age at the time of the interview, federal state dummies, a female indicator, survey and interview year dummies, and federal state-specific time trends, i.e., interactions of school state dummies with a linear trend in year of birth. We cluster standard errors at the state*×*year of birth levels in all specifications. The coefficients of interest, $\beta _g$, are reported in Fig. 1.

The results show a favorable correlation between education and health over the life cycle. An additional year of schooling is correlated with a lower probability of having been hospitalized in the previous year, a lower number of diagnosed (chronic) diseases, a lower probability of rating one’s own health as poor or bad, and a lower BMI at all ages. Although the coefficients are small in absolute terms, they are sizable when compared to the sample means in Table 2. For example, the coefficient for hospitalization hovers around -0.01 (especially for older individuals). The sample mean is 0.12, implying an 8% lower probability of hospitalization per year of education compared to the mean. The evolution of the coefficients differs for each outcome over the life cycle. There is no clear pattern for hospitalization, but the education-health gap (comparing individuals with more versus less education) appears to be slightly larger for older ages. The same applies to BMI. The largest gradients for the number of diagnosed illnesses exist between the ages of 55 and 65. Regarding self-reported poor or bad health, educational differences increase up until age 60, then decrease for the oldest age groups.

## Instrumental variables estimations by age group

### Empirical strategy

We estimate the effect of education on health using two-stage least squares (2SLS) in a fuzzy regression discontinuity design framework. Specifically, we run the following regressions:

2 $$\begin{aligned} H_{ist}= & \sum _{g}{\beta _g Yed_{is} \times agegroup_{it}} + \gamma _1 \widetilde{bc}_{is} + \gamma _2 \widetilde{bc}_{is}\nonumber \\ & \times Reform_{is} + \beta X_{ist} + \varepsilon _{ist} \end{aligned}$$

where $Yed_{is} \times agegroup_{it}$ is instrumented by $Reform_{is} \times agegroup_{it}$.^3^$Reform_{is}$ is an indicator variable for whether an individual was affected by a compulsory schooling reform in school state *s* or not, i.e., whether they were born in or after the pivotal cohort (see Table 1 above). $\widetilde{bc}_{is}$ is the normalized birth cohort (the distance between the year of birth and the relevant pivotal cohort) that is, the running variable. Control variables in $X_{ist}$ are a full set of fixed effects for age, federal state of birth fixed, survey year, data source, and gender.

We provide estimates of the relevant first stages in Fig. 9 in the Appendix to show that the compulsory schooling reform increased educational attainment across all cohorts and subsamples (identified by data availability for our different outcome variables). Figure 9 also presents the first-stage F-statistics of the excluded instrument (again, separate by age group) to demonstrate our instrument strength across the life cycle and outcomes. Except for one coefficient the first-stage F-statistics are above the commonly used threshold of 10.^4^

For a causal interpretation, the cutoff at the pivotal cohorts must be exogenous to the individuals in our sample. This implies that only compulsory schooling changes at the cutoff, so that individuals immediately to the left and right of the cutoff are comparable. The inclusion of state-specific cohort trends as controls supports the validity of this assumption, as these trends help to control for any factors that may have affected cohorts differently in different states. Another reform that occurred concurrently with compulsory education reforms in some states was a shift in the start of the school year from spring to fall. In 1966-1967, most West German states, with the exception of Bavaria, where the school year had already started in the fall, introduced two short school years to accommodate this shift [18]. These short school years had only 24 weeks of instruction compared to the usual 37 weeks. This had the effect that students formally completed nine years of schooling despite having received little additional instruction compared to earlier cohorts. This could potentially affect the identifying assumption and/or bias the estimates. To address both, we show a robustness check where we include indicator variables for short school years. The estimates do not differ from those obtained in our main results. This is in line with the literature, in particular [19], who show that the estimates are generally robust to these adjustments. In Baden-Württemberg, Hesse, North Rhine-Westphalia and Rhineland-Palatinate, the short school years coincided with the introduction of compulsory schooling reforms [18]. In another robustness check, we remove these states from the analysis. This does not change the results.

The compulsory schooling reform has been widely studied, but potential spillover or general equilibrium effects are rarely addressed. If the reform indirectly affected individuals outside the treated cohorts – such as those born just before or in states yet to adopt the reform – who are used in the control group, the estimates may not reflect the true effect. Schiele [27] studies labor market spillovers from the reform, as delaying an entire cohort’s entry into the labor market could affect opportunities for untreated individuals. He finds no long-run employment or wage effects for men but some effects for women. Below we also generate effects separately by men and women but do not find substantial differences.

### Estimation results

**Fig. 2:**
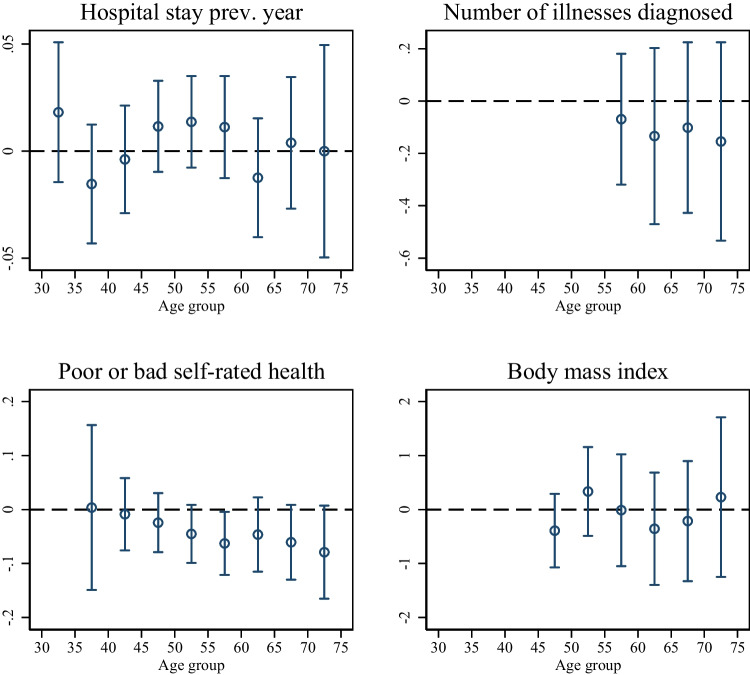
Two-stage least squares results. *Notes:* This figure shows estimates of the effect of years of schooling on four health outcomes across up to nine age groups. Hospital stay is an indicator variable for whether a person was hospitalized for at least one night in the past year. Number of diagnoses is a count of the total number of chronic conditions and diseases diagnosed from a list of thirteen possible options. Poor or bad self-rated health is an indicator variable for selecting one of the two lowest of the five possible self-rated health categories. BMI is weight (in kilograms) divided by height squared (in meters). Own calculations based on data from SOEP and our sample selection, as outlined in “Institutional framework and data” section. We visualize point estimates of coefficients $\beta _g$ based on 2SLS (2) alongside 95% confidence intervals. Instruments are the interactions between the reform dummy variable (pivotal cohort and older) and the age groups. Coefficients plotted between 30 and 35 represent age group 30-34, and so on. Some age groups are missing due to data constraints. Standard errors clustered at state*×*year of birth level

Figure 2 shows our main results from the second stage of the 2SLS estimations. For all outcomes, the effect of an additional year of schooling fluctuates around zero over the life cycle. This pattern provides evidence that the positive correlation between education and health shown in Fig. 1 is unlikely to represent a causal effect of education. Some age-group-specific point estimates of hospitalizations and BMI are below, some are above zero. For diagnoses and self-rated health, all coefficients are either exactly zero or have a negative sign. In general, all estimates are very close to zero and not statistically significant. A possible exception may be poor or bad self-assessed health, where the estimates point towards a null effect, but are increasing in age. The coefficients for the oldest age groups are relevant in size, but not statistically significant. We investigate further and in the “A simulated ex-post power analysis” section show that this does not seem to be due to an underpowered sample. Our power analysis reveals that we would theoretically be able to detect significant effects on poor or bad self-rated health if they are above *-2.6* to *-3* percentage points, depending on the age group.

**Fig. 3:**
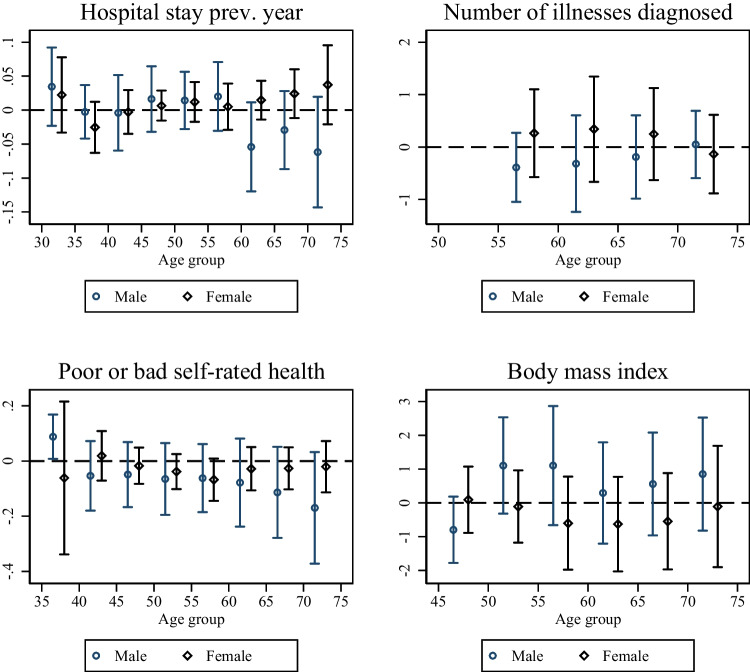
Heterogeneous effects by gender. *Notes:* This figure shows estimates of the effect of years of schooling on four health outcomes across up to nine age groups by gender. Hospital stay is an indicator variable for whether a person was hospitalized for at least one night in the past year. Number of diagnoses is a count of the total number of chronic conditions and diseases diagnosed from a list of thirteen possible options. Poor or bad self-rated health is an indicator variable for selecting one of the two lowest of the five possible self-rated health categories. BMI is weight (in kilograms) divided by height squared (in meters). Own calculations based on data from SOEP and our sample selection, as outlined in “Institutional framework and data” section. We visualize point estimates of coefficients $\beta _g$ based on 2SLS (2) alongside 95% confidence intervals. Instruments are the interactions between the reform dummy variable (pivotal cohort and older) and the age groups. Coefficients plotted between 30 and 35 represent age group 30-34, and so on. Some age groups are missing due to data constraints. Standard errors clustered at state*×*year of birth level

Figure 3 shows heterogeneous effects by gender. We find no structural differences between men and women for the first three outcomes. Point estimates on diagnoses are positive for women in most age groups and negative for men. Conversely, point estimates on BMI are negative for women and positive for men across most age groups. Nevertheless, all are close to zero and not statistically significant.

In summary, the main findings of the paper are as follows: individuals with more schooling are in better health, and the health gap by education increases until late midlife for some outcomes. However, there is no evidence of a causal local average treatment effect of additional schooling for individuals with low educational margins. We do not observe broad improvements in health due to the additional year of schooling from the German compulsory schooling reforms up to age 74. There are indications that there may be an effect of relevant size for self-assessed health. However, the coefficients are not statistically significant.

How do our results relate to the previous literature on age-specific effects of schooling? Our results are consistent with [10], who find no effects of education on mortality over the life cycle using the British schooling reform of 1947. This reform increased the minimum school-leaving age from 14 to 15. While certainly different in many details, it shares a similarity with the German one that increased years of compulsory schooling from 8 to 9 (effectively shifting the school-leaving age from 14 to 15 as well). Clark and Royer [10] also find no mortality effects of the 1972 reform, which increased the minimum school-leaving age from 15 to 16. In contrast, Gehrsitz and Williams Jr [11] report some effects of that reform on hospitalizations. In discussing why the two British reforms yielded different results, they emphasize differences in the groups of affected compliers. Referring to [28], they argue that the 1947 reform “overwhelmingly affected students in lower-track schools” — precisely the group also targeted by the German reform—whereas the 1972 reform influenced students at a different educational margin. In their online appendix, Gehrsitz and Williams Jr [11] also present evidence of weak effects of the 1947 reform, though without distinguishing by age. Taken together, this suggests that our findings are closely aligned with the evidence from the British 1947 reform, which—unlike the 1972 reform—is comparable to the German case in terms of the affected population.

### Robustness checks

We perform several robustness checks and report the results in the Appendix. To corroborate our main findings, we first present reduced-form results in Fig. 10. It shows estimates of the instrument (being born in or after a pivotal cohort) and its interactions with age groups on our four outcomes. Our main results are also present in the reduced form. The effects have a similar pattern. In Fig. 11 we repeat the baseline estimation but do not account for state-specific cohort trends. In Fig. 12 we control for short school years by adding an indicator variable for cohorts that were affected. In Fig. 13 we exclude the states for which short school years coincided with the introduction of the compulsory schooling reform. Based on both, we can rule out that short school years affected our results (also see our point in “Empirical strategy” section).

In Figs. 14, 15 and 16, we provide robustness checks for a different sample selection. Instead of choosing a bandwidth of five years around the pivotal cohort in each state we use four, six and seven years. The patterns remain almost identical. The only exception is the marginally statistically significant effect of education on hospitalization between the ages of 45 and 60, when the bandwidth is *± 6* years. However, there are no consistent differences in patterns.

In Fig. 17 we show results using the established state cutoffs from [16] to demonstrate that – while the cutoffs from [22] reflect the institutional setting at the time more accurately – our results do not rely on them. Finally, in Fig. 18 we show our main results for an alternative measure of the number of diagnoses where we exclude the “other illnesses” category, since it may include unknown or less severe illnesses that might not fit our intended interpretation. This category contributes to the diagnoses count of 17 percent of the observations in the respective sample. Nevertheless, the results are virtually identical when excluding it. Overall, our results are robust to all aforementioned checks. They do not differ qualitatively from our main results.

### A simulated ex-post power analysis

How likely is it that the true effect of education on health is economically significant, yet we fail to reject the null hypothesis of no effect due to a too small sample? This question may be relevant to our analysis of self-rated health. While the estimates here are statistically insignificant, the coefficients of the oldest age groups may be economically relevant. This cannot be said for the other three outcomes, for which the statistical insignificance is likely driven by small coefficients (especially in relation to the sample mean) rather than large standard errors. To assess statistical power for the analysis on self-rated health, we adopt a simulation-based approach from [12] and identify the minimum detectable effect sizes (MDEs).^5^ The MDE represents the minimum effect size with 80% power at the 5% significance level. In other words, we are looking for an estimate that, if it were the true effect, would lead us to reject the null hypothesis of no effect at the 5% significance level in 80% of all simulated cases. To simplify the analysis, we focus on the reduced-form relationship. That is, we estimate the direct effect of being affected by the compulsory schooling reform on the probability of rating one’s health as poor or bad in the four oldest age groups. Nevertheless, we can relate the results back to our 2SLS results by dividing the MDEs by the respective relevant first stages (see Fig. 9). By only considering the reduced form in this analysis, we do not need further assumptions on how individuals will react to the reform, i.e., whether they are compliers or always takers.

In short, we randomly assign treatment status and an artificial treatment effect to observations unaffected by the schooling reform and run 1000 regressions for each artificial treatment effect (stepwise increased from 0.001 and 0.03) to check at which size we reject the null hypothesis of no effect in 80% of all regressions. The detailed steps involved in the simulated power analysis are described in Appendix B.

**Fig. 4:**
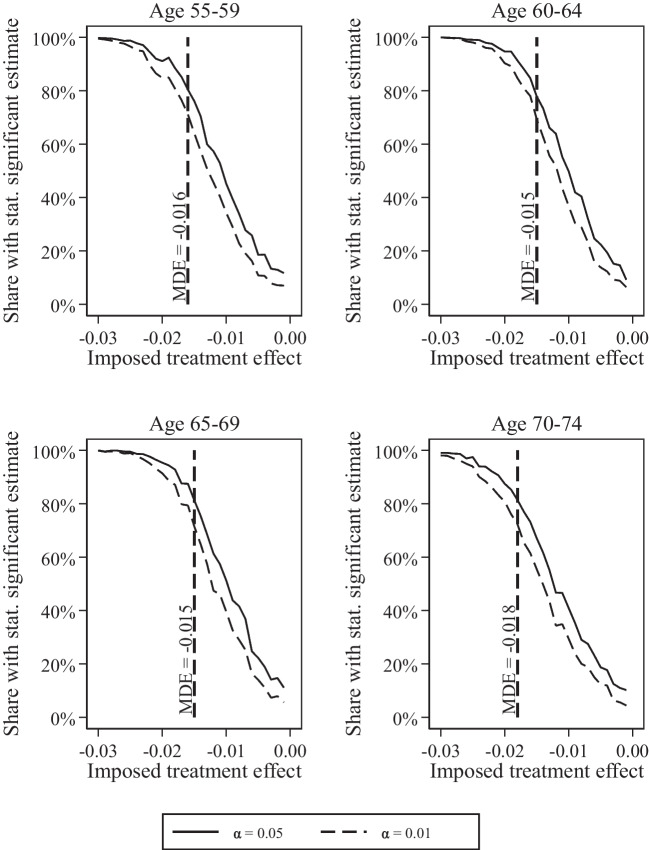
Simulated power analysis – *Poor or bad self-rated health*. *Notes:* This figure shows the proportion of simulated cases in which we would reject the null hypothesis of no effect of the compulsory schooling reform on poor or bad self-rated health at the 5% level, given a simulated, hypothetical treatment effect for each age group. We visualize the minimum detectable effect sizes (MDEs) as vertical dashed lines, which refers to the effect size at which we would reject the null hypothesis of no effect at the 5% significance level in 80% of all simulated cases. The simulations are based on [12]. Treatment status is randomly assigned to pre-treatment observations, matching the original sample size and school-state distributions. A synthetic treatment effect is imposed on the treated observations. We run 1,000 repetitions of reduced-form regressions, marginally increasing the treatment effect size each time from 0.001 to 0.03 in steps of 0.001

Figure 4 reports the results of this exercise. The MDE is shown as a vertical dashed line at the point where we detect a statistically significant effect at the 5% level in 800 of the 1,000 simulations. The MDEs are small and do not differ substantially between age groups. Given our setting, sample, and data structure, we would in theory be able to reliably detect true treatment effects of the compulsory schooling reform on poor or bad self-rated health if they are at least between *-1.5* and *-1.8* percentage points in size, depending on the age group. Dividing each groups-specific reduced-form MDE by their respective first stage yields 2SLS MDEs of *-2.9* percentage points for 55–59 year-olds, *-2.6* for 60–64 and 65–69 year-olds, and *-3* percentage points for 70–74 year-olds.^6^ We interpret these findings as evidence that our main results seem to have enough power. Even though our analysis finds relatively sizable, but statistically insignificant estimates for self-rated health, we cannot rule out that the true effect is zero.

We also investigate power in the BMI analysis sub-sample for these four age groups where we have substantially less observations per group. Figure 19 in the appendix shows the results. Even with this much smaller sample size, we would be able to reliably identify true reduced-form effects of the schooling reform on BMI if they were at least between *-0.129* and $-0.186 \text { kg/m}^2$. The 2SLS MDEs in the four age groups are between *-0,237* and *-0.31*, meaning we would be able to pick up on very small effects, especially when compared to the mean BMI of $26.74 \text { kg/m}^2$.

### Attrition

A concern with longitudinal household surveys, especially those focused on older populations and where health is of interest, is potential bias due to attrition [30–32]. Attrition hinders a survey from being representative of the target population and can create barriers to statistical inference [30, 31]. Although the surveys we use are constantly updated, selective attrition (e.g. due to mortality) by educational status can be a problem.

We use two complementary approaches to test for potential attrition problems. First, we create a binary indicator *attrition* in our working sample. This indicator equals one if an individual does not appear in the next wave of the survey and zero if they either appear in the next wave or if it is the last wave (year 2022) of the survey.

**Fig. 5:**
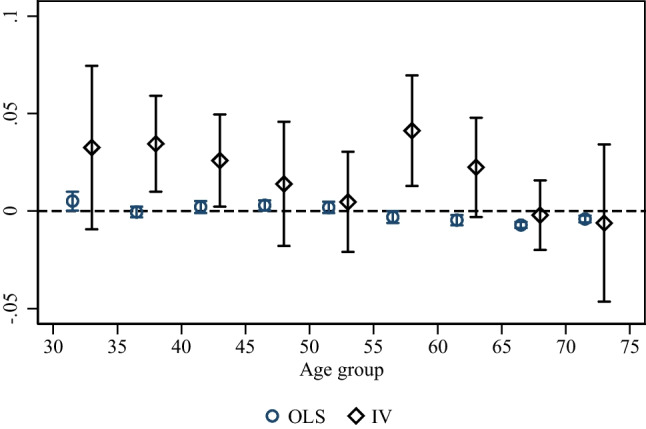
Effect of education on attrition over the life-cycle. *Notes:* This figure shows estimates of the effect of years of schooling on attrition across nine age groups. Attrition is an indicator of leaving the sample in a given wave after being in the sample in the previous wave. Own calculations based on data from SOEP and our sample selection, as outlined in “Institutional framework and data” section. We visualize point estimates alongside 95% confidence intervals. Coefficients plotted between 30 and 35 represent age group 30-34, and so on. Standard errors clustered at state *×* year of birth

We generate this indicator before selecting the sample based on the pivotal cohort. By this definition, 20 percent of all person-year observations in our sample drop out between two waves. Next, we use *attrition* as the outcome and run OLS and IV regressions as before. Figure 5 shows the results of this exercise.

The OLS estimates suggest no relationship between educational attainment and attrition. Our 2SLS estimates point to a small increase in sample attrition due to education for compliers in the youngest age groups as well as for groups 55-60 and 60-65 and a null effect for 50-55 and the oldest age groups. Yet, all differences are very small.

As a second approach, we reduce the sample to one observation per individual and create indicator variables if they are still in our original sample at ages 50, 60, 65, 70 or 75. For example, for a person born in 1930 who drops out of the sample in 2001, all indicator variables except the one for age 75 would be set to one. If a person is still in the sample in 2021 and has not reached one of the age thresholds, the respective indicators are set to missing.

**Fig. 6:**
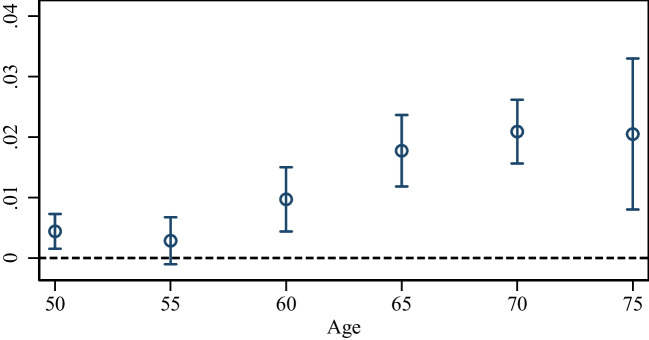
Relationship of education on (still) being in the sample at certain ages. *Notes:* This figure shows estimates of years of schooling on the likelihood of still being in the sample at specific ages between 50 and 75. Own calculations based on data from SOEP and our sample selection, as outlined in “Institutional framework and data” section. We visualize point estimates alongside 95% confidence intervals. Standard errors clustered at state *×* year of birth

87% of all individuals are still in the survey at age 50 (based on 15,228 individuals). This number drops steadily to 43% who are still in the survey at age 75 (based on 1,592 individuals). We then run separate regressions of all indicators on years of schooling, female, year of birth dummies, survey and state dummies, and state-specific linear cohort trends. The results are shown in Fig. 6. We observe a higher probability of remaining in the sample at older ages with more education. The differences increase with age and are statistically significant, but very small in absolute terms. Individuals with one more year of schooling are on average 2.4 percentage points more likely to still be in our sample at age 75. We take the results as indication that selective attrition is unlikely to have a significant effect on our regression results.

## Effects on non-health outcomes

One reason we do not find clear and consistent causal health effects of compulsory schooling reform in Germany, even in the long run, may be the absence of effects on other outcomes that could have influenced later-life health. The strongest possibilities are certainly labor market effects, health behavior, or determinants of general decision-making such as risk preferences.

Labor market effects on health could come in two flavors: through income or through healthier jobs. The original analysis from [16] finds zero wage returns of this reform and suggests that the institutional setting itself may be to blame. They argue that basic skills of the compliers, which are necessary for the labor market, may already be settled after eight years of schooling and/or the additional schooling did not further improve them. This point is investigated and corroborated by [20], who, using data from SOEP, find that this reform had no effect on cognitive abilities later in life. However, a reanalysis of [16], offers evidence of wage effects [14].

**Fig. 7:**
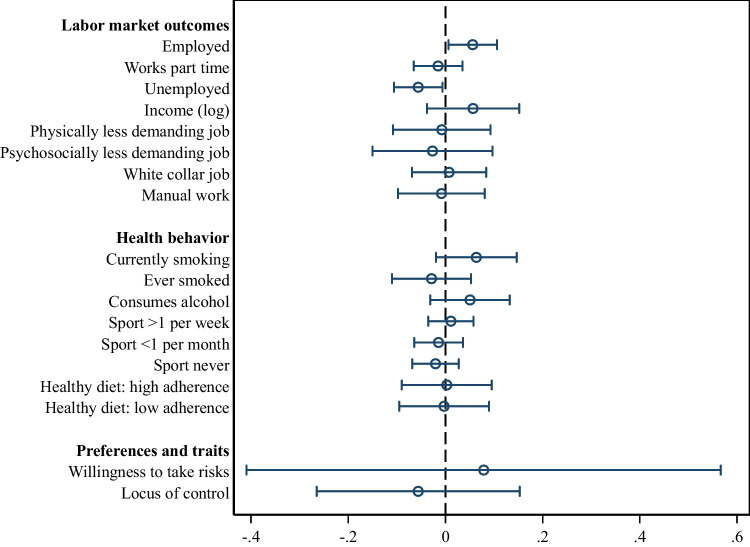
Effects of education on additional non-health outcomes. *Notes:* The figure shows instrumented 2SLS estimates of years of schooling on several non-health outcomes alongside 95% confidence intervals. Own calculations based on data from SOEP and our sample selection, as outlined in “Institutional framework and data” section. All outcomes are binary, except willingness to take risks (which is a scale from 0 to 10) and locus of control (which is a standardized factor variable). Therefore, confidence intervals on these variables seem a lot larger when displayed next to the confidence bands from the binary variables. For details on all outcomes, see Appendix C. Years of schooling is instrumented with whether the individual was subject to a federal-state-specific compulsory schooling reform. Controls include a gender dummy as well as age, state, survey and survey year fixed effects as well as normalized birth cohort and normalized birth cohort *× Reform*. Standard errors clustered at state*×*year of birth level

**Fig. 8:**
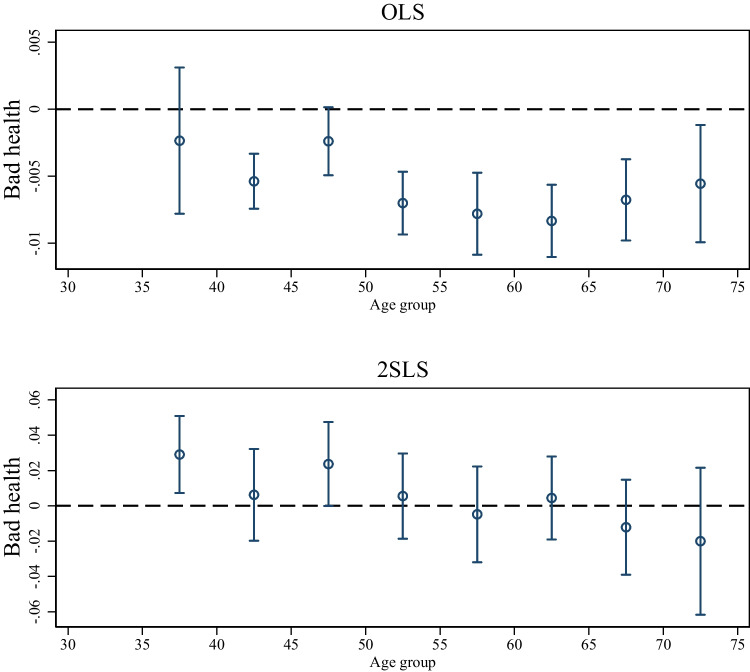
Additional outcomes – *Bad health*. *Notes:* This figure shows estimates of the effect of years of schooling on self-reported bad health across eight age groups. Bad health is a binary indicator of rating the own current health condition as bad. Own calculations based on data from SOEP and our sample selection, as outlined in “Institutional framework and data” section. We visualize point estimates of coefficients $\beta _g$ based on 2SLS (2) alongside 95% confidence intervals. Instruments are the interactions between the reform dummy variable (pivotal cohort and older) and the age groups. Coefficients plotted between 30 and 35 represent age group 30-34, and so on. Some age groups are missing due to data constraints. Standard errors clustered at state*×*year of birth level

**Fig. 9:**
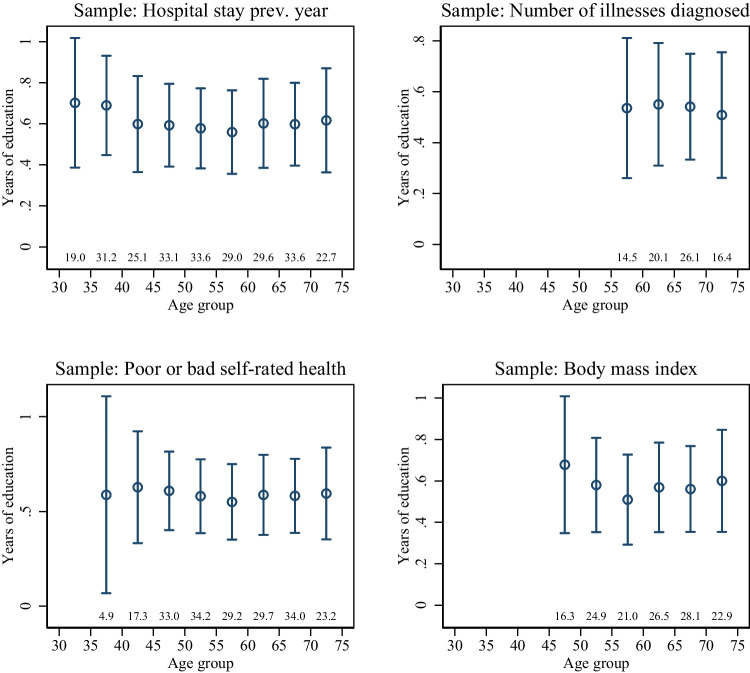
First stages. *Notes:* This figure shows first stage results of our main specification, i.e., point estimates and 95% confidence intervals of the coefficients of *Reform × agegroup* in the first stage regressions that corresponds to Eq. 2. The numbers below are first-stage F-statistics of the excluded relevant instrument. The relevant instrument is the age group interaction with the reform dummy that corresponds to the second stage interaction with school years. For example, in the upper left panel, the relevant instrument for $Schoolyears \times age group_{30-34}$ is $Reform \times age group_{30-34}$, with corresponding F-statistic of 19.0. Own calculations based on data from SOEP and our sample selection, as outlined in “Institutional framework and data” section. Coefficients plotted between 30 and 35 represent age group 30-34, and so on. Some age groups are missing due to data constraints. Standard errors clustered at state*×*year of birth level

**Fig. 10:**
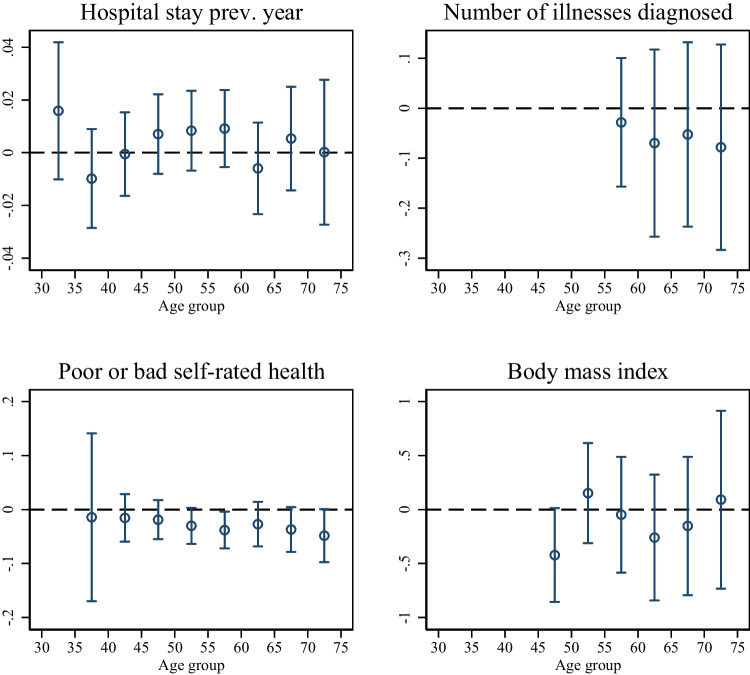
Reduced-form effects. *Notes:* This figure shows reduced form effects, i.e., the direct effect of compulsory schooling reforms on four health outcomes for up to nine age groups. Hospital stay is an indicator variable for whether a person was hospitalized for at least one night in the past year. Number of diagnoses is a count of the total number of chronic conditions and diseases diagnosed from a list of thirteen possible options. Poor or bad self-rated health is an indicator variable for selecting one of the two lowest of the five possible self-rated health categories. BMI is weight (in kilograms) divided by height squared (in meters). Own calculations based on data from SOEP and our sample selection, as outlined in “Institutional framework and data” section. We visualize coefficients of interactions between the instrument (a dummy for being born in or after the key cohort of a compulsory schooling reform) and the age group alongside 95% confidence intervals. Coefficients plotted between 30 and 35 represent age group 30-34, and so on. Some age groups are missing due to data constraints. Standard errors clustered at state*×*year of birth level

**Fig. 11:**
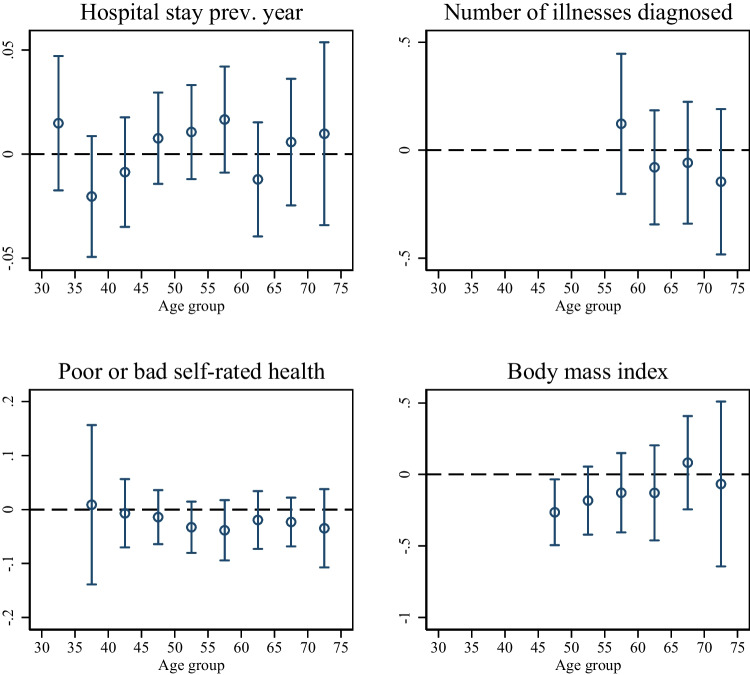
Robustness – *No state* *×* *cohort trends*. *Notes:* This figure shows a robustness check of our main results. We estimate the causal effect of years of schooling on four health outcomes across up to nine age groups. Here, we omit state-specific cohort trends that we usually control for. Hospital stay is an indicator variable for whether a person was hospitalized for at least one night in the past year. Number of diagnoses is a count of the total number of chronic conditions and diseases diagnosed from a list of thirteen possible options. Poor or bad self-rated health is an indicator variable for selecting one of the two lowest of the five possible self-rated health categories. BMI is weight (in kilograms) divided by height squared (in meters). Own calculations based on data from SOEP and our sample selection, as outlined in “Institutional framework and data” section. We visualize point estimates of coefficients $\beta _g$ based on 2SLS (2) alongside 95% confidence intervals. Instruments are the interactions between the reform dummy variable (pivotal cohort and older) and the age groups. Coefficients plotted between 30 and 35 represent age group 30-34, and so on. Some age groups are missing due to data constraints. Standard errors clustered at state*×*year of birth level

**Fig. 12:**
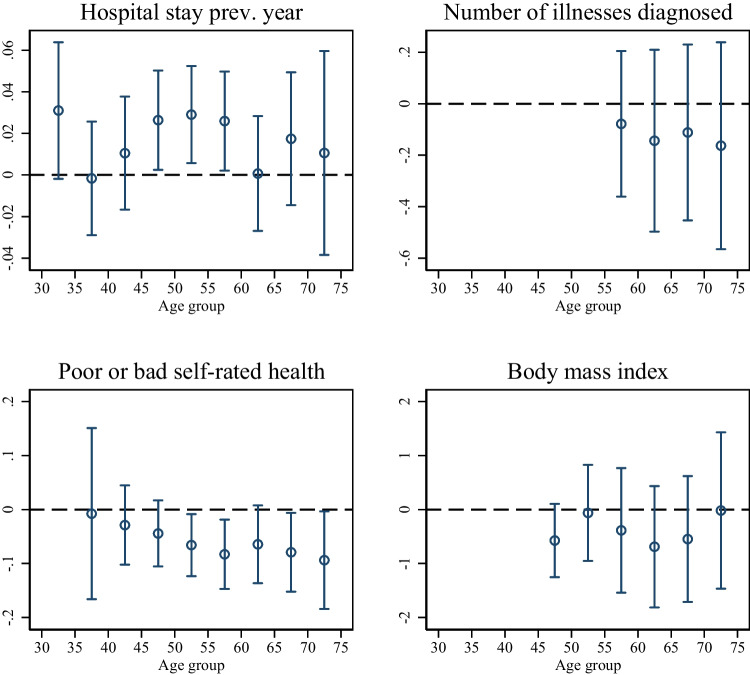
Robustness – *Controlling for short school years*. This figure shows a robustness check of our main results. We estimate the causal effect of years of schooling on four health outcomes across up to nine age groups but add an indicator variable for cohorts who were exposed to short school years. Between 1966 and 1967, the introduction of two short school years in West Germany to adjust the academic calendar coincided with some compulsory schooling reforms and may have overstated formal schooling for some cohorts. Hospital stay is an indicator variable for whether a person was hospitalized for at least one night in the past year. Number of diagnoses is a count of the total number of chronic conditions and diseases diagnosed from a list of thirteen possible options. Poor or bad self-rated health is an indicator variable for selecting one of the two lowest of the five possible self-rated health categories. BMI is weight (in kilograms) divided by height squared (in meters). Own calculations based on data from SOEP and our sample selection, as outlined in “Institutional framework and data” section. We visualize point estimates of coefficients $\beta _g$ based on 2SLS (2) alongside 95% confidence intervals. Instruments are the interactions between the reform dummy variable (pivotal cohort and older) and the age groups. Coefficients plotted between 30 and 35 represent age group 30-34, and so on. Some age groups are missing due to data constraints. Standard errors clustered at state*×*year of birth level

**Fig. 13:**
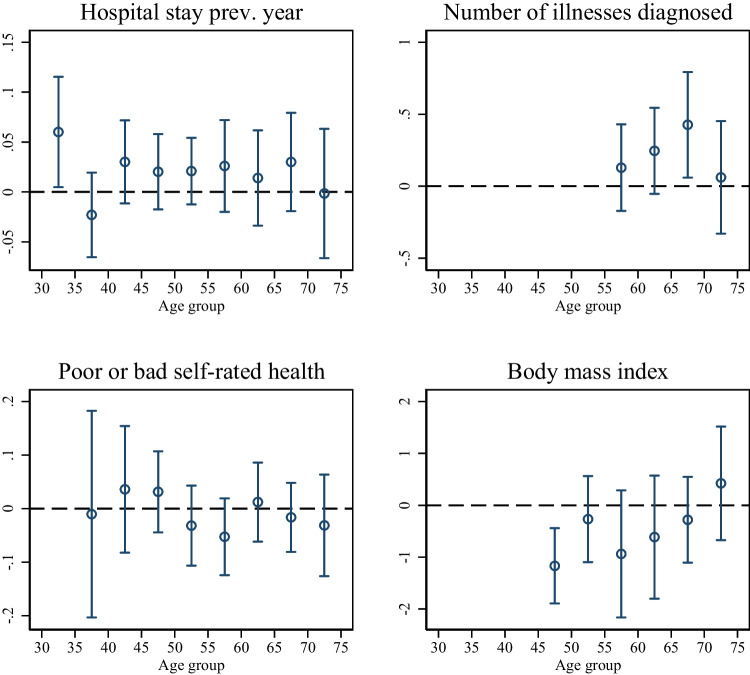
Robustness – *Excluding states with simultaneous reform and short years*. *Notes:* This figure shows a robustness check of our main results. We estimate the causal effect of years of schooling on four health outcomes across up to nine age groups. However, we remove the states of Baden-Württemberg, Hesse, North Rhine-Westphalia, and Rhineland-Palatinate from the analysis, as the introduction of compulsory schooling coincided with a shortened school year in these states. Hospital stay is an indicator variable for whether a person was hospitalized for at least one night in the past year. Number of diagnoses is a count of the total number of chronic conditions and diseases diagnosed from a list of thirteen possible options. Poor or bad self-rated health is an indicator variable for selecting one of the two lowest of the five possible self-rated health categories. BMI is weight (in kilograms) divided by height squared (in meters). Own calculations based on data from SOEP and our sample selection, as outlined in “Institutional framework and data” section. We visualize point estimates of coefficients $\beta _g$ based on 2SLS (2) alongside 95% confidence intervals. Instruments are the interactions between the reform dummy variable (pivotal cohort and older) and the age groups. Coefficients plotted between 30 and 35 represent age group 30-34, and so on. Some age groups are missing due to data constraints. Standard errors clustered at state*×*year of birth level

**Fig. 14:**
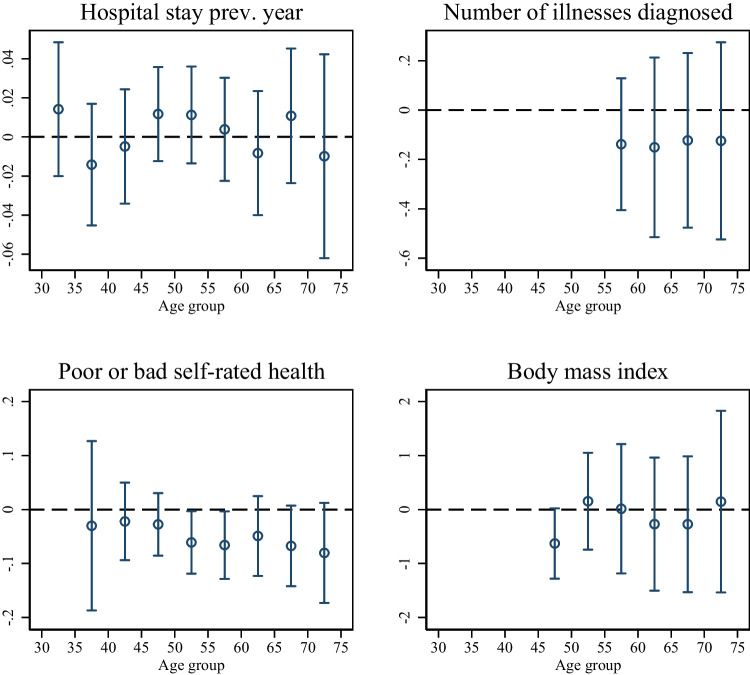
Robustness – *Bandwidth of 4 years*. *Notes:* This figure shows a robustness check of our main results. We estimate the causal effect of years of schooling on four health outcomes across up to nine age groups. Here, instead of selecting our sample to be *± 5* cohorts around each pivotal cohort of the education reform, we use *± 4*. Hospital stay is an indicator variable for whether a person was hospitalized for at least one night in the past year. Number of diagnoses is a count of the total number of chronic conditions and diseases diagnosed from a list of thirteen possible options. Poor or bad self-rated health is an indicator variable for selecting one of the two lowest of the five possible self-rated health categories. BMI is weight (in kilograms) divided by height squared (in meters). Own calculations based on data from SOEP and our sample selection, as outlined in “Institutional framework and data” section. We visualize point estimates of coefficients $\beta _g$ based on 2SLS (2) alongside 95% confidence intervals. Instruments are the interactions between the reform dummy variable (pivotal cohort and older) and the age groups. Coefficients plotted between 30 and 35 represent age group 30-34, and so on. Some age groups are missing due to data constraints. Standard errors clustered at state*×*year of birth level

**Fig. 15:**
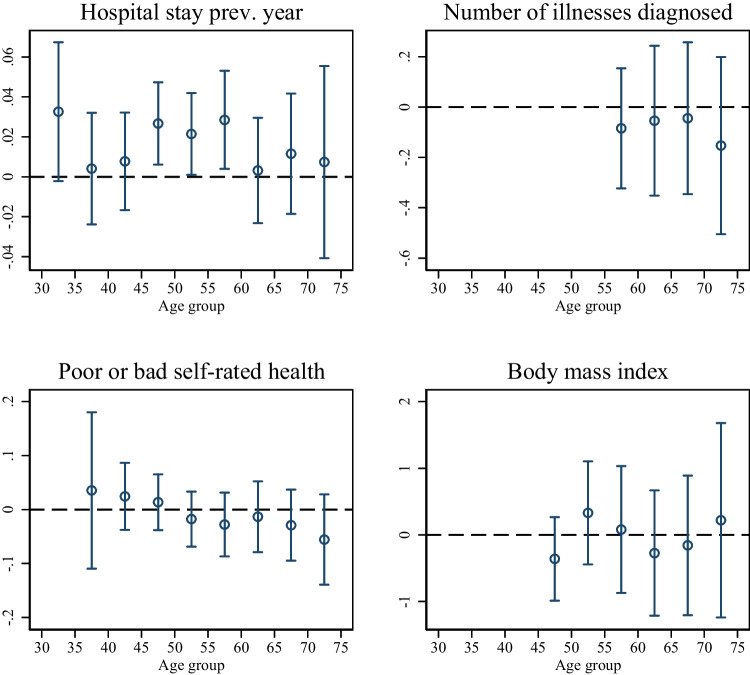
Robustness – *Bandwidth of 6 years*. *Notes:* This figure shows a robustness check of our main results. We estimate the causal effect of years of schooling on four health outcomes across up to nine age groups. Here, instead of selecting our sample to be *± 5* cohorts around each pivotal cohort of the education reform, we use *± 6*. Hospital stay is an indicator variable for whether a person was hospitalized for at least one night in the past year. Number of diagnoses is a count of the total number of chronic conditions and diseases diagnosed from a list of thirteen possible options. Poor or bad self-rated health is an indicator variable for selecting one of the two lowest of the five possible self-rated health categories. BMI is weight (in kilograms) divided by height squared (in meters). Own calculations based on data from SOEP and our sample selection, as outlined in “Institutional framework and data” section. We visualize point estimates of coefficients $\beta _g$ based on 2SLS (2) alongside 95% confidence intervals. Instruments are the interactions between the reform dummy variable (pivotal cohort and older) and the age groups. Coefficients plotted between 30 and 35 represent age group 30-34, and so on. Some age groups are missing due to data constraints. Standard errors clustered at state*×*year of birth level

**Fig. 16:**
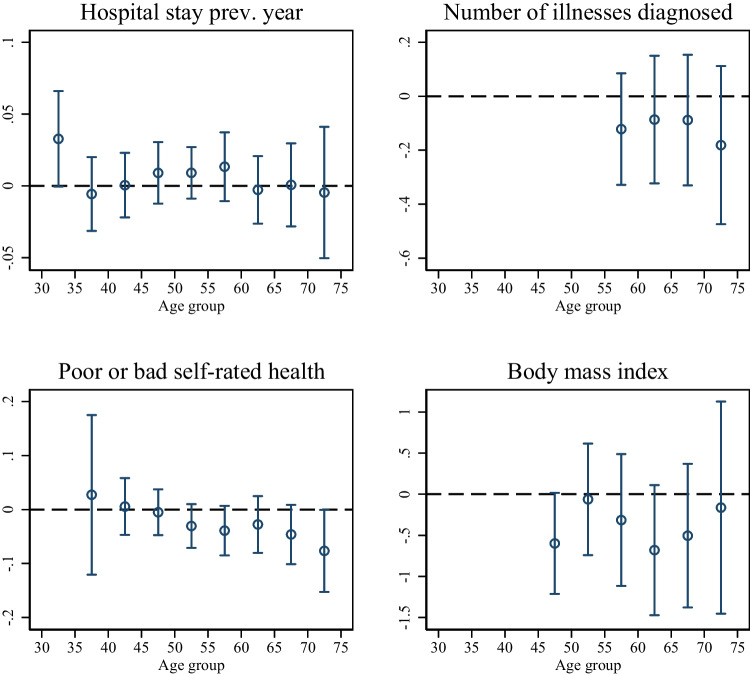
Robustness – *Bandwidth of 7 years*. *Notes:* This figure shows a robustness check of our main results. We estimate the causal effect of years of schooling on four health outcomes across up to nine age groups. Here, instead of selecting our sample to be *± 5* cohorts around each pivotal cohort of the education reform, we use *± 7*. Hospital stay is an indicator variable for whether a person was hospitalized for at least one night in the past year. Number of diagnoses is a count of the total number of chronic conditions and diseases diagnosed from a list of thirteen possible options. Poor or bad self-rated health is an indicator variable for selecting one of the two lowest of the five possible self-rated health categories. BMI is weight (in kilograms) divided by height squared (in meters). Own calculations based on data from SOEP and our sample selection, as outlined in “Institutional framework and data” section. We visualize point estimates of coefficients $\beta _g$ based on 2SLS (2) alongside 95% confidence intervals. Instruments are the interactions between the reform dummy variable (pivotal cohort and older) and the age groups. Coefficients plotted between 30 and 35 represent age group 30-34, and so on. Some age groups are missing due to data constraints. Standard errors clustered at state*×*year of birth level

**Fig. 17:**
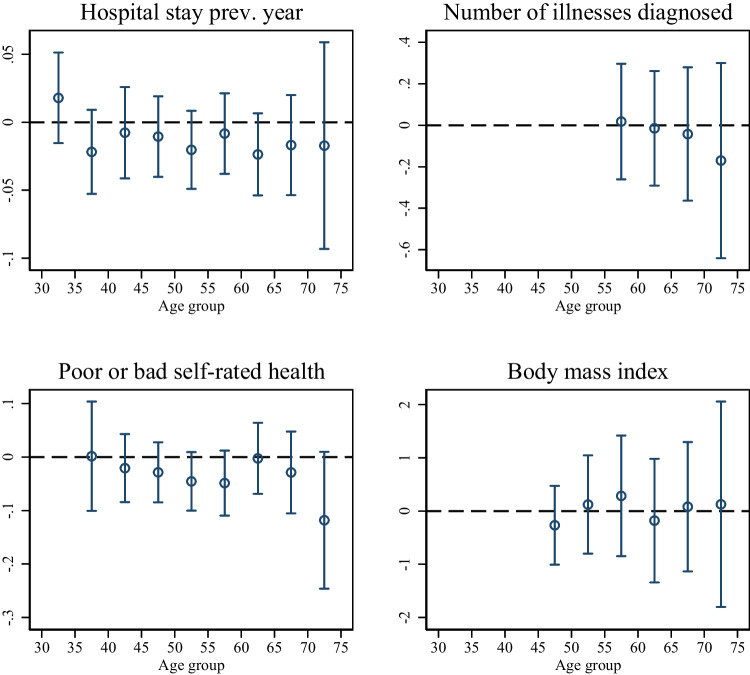
Robustness – *Cutoffs from* [16]. *Notes:* This figure shows a robustness check of our main results. We estimate the causal effect of years of schooling on four health outcomes across up to nine age groups using the well-established compulsory schooling state cutoffs from [16]. Hospital stay is an indicator variable for whether a person was hospitalized for at least one night in the past year. Number of diagnoses is a count of the total number of chronic conditions and diseases diagnosed from a list of thirteen possible options. Poor or bad self-rated health is an indicator variable for selecting one of the two lowest of the five possible self-rated health categories. BMI is weight (in kilograms) divided by height squared (in meters). Own calculations based on data from SOEP and our sample selection, as outlined in “Institutional framework and data” section. We visualize point estimates of coefficients $\beta _g$ based on 2SLS (2) alongside 95% confidence intervals. Instruments are the interactions between the reform dummy variable (pivotal cohort and older) and the age groups. Coefficients plotted between 30 and 35 represent age group 30-34, and so on. Some age groups are missing due to data constraints. Standard errors clustered at state*×*year of birth level

**Fig. 18:**
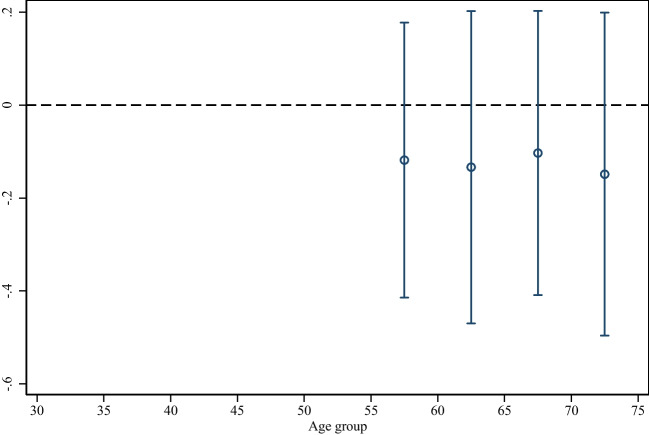
Robustness – *Number of illnesses excluding ‘other illnesses’*. *Notes:* This figure shows a robustness check of our main results. We estimate the causal effect of years of schooling on the number of diagnoses, but when counting up the illnesses disregard the category “other illnesses”. Own calculations based on data from SOEP and our sample selection, as outlined in “Institutional framework and data” section. We visualize point estimates of coefficients $\beta _g$ based on 2SLS (2) alongside 95% confidence intervals. Instruments are the interactions between the reform dummy variable (pivotal cohort and older) and the age groups. Coefficients plotted between 30 and 35 represent age group 30-34, and so on. Some age groups are missing due to data constraints. Standard errors clustered at state*×*year of birth level

**Fig. 19:**
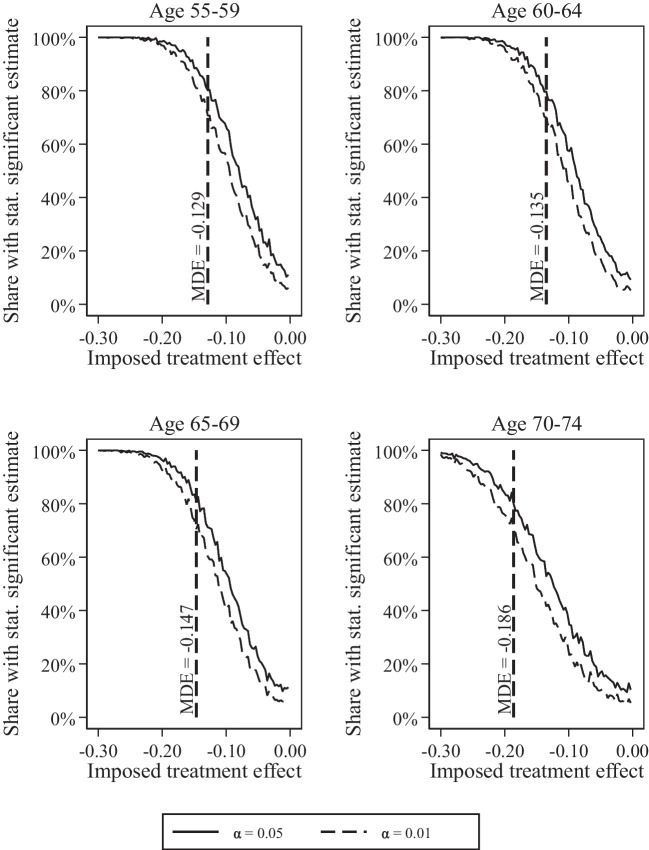
Simulated power analysis – *Body mass index*. *Notes:* This figure shows the proportion of simulated cases in which we would reject the null hypothesis of no effect of the compulsory schooling reform on BMI at the 5% level, given a simulated, hypothetical treatment effect for each age group. We visualize the minimum detectable effect sizes (MDEs) as vertical dashed lines, which refers to the effect size at which we would reject the null hypothesis of no effect at the 5% significance level in 80% of all simulated cases. The simulations are based on [12]. Treatment status is randomly assigned to pre-treatment observations, matching the original sample size and school-state distributions. A synthetic treatment effect is imposed on the treated observations. We run 1,000 repetitions of reduced-form regressions, marginally increasing the treatment effect size each time from 0.003 to 0.3 in steps of 0.003

Our additional analyses in Fig. 7 suggest a small, statistically insignificant estimate of the reform on labor income as well as a statistically significant positive effect of the additional year of schooling on the likelihood of being employed and a significant negative effect on the likelihood of being unemployed. Our wage point estimate (a 5.7 percent increase due to the additional year) is comparable in size to [14], albeit not statistically significant.^7^ Apart from an already established skill set, another reason we do not see health effects from compulsory schooling could be that the additional schooling does not lead to healthier jobs. We investigate this using four classifications of occupations pertaining to healthier jobs: white-collar versus blue-collar, physically demanding versus less demanding, psychosocially demanding versus less demanding, and manual versus non-manual.^8^ In our sample, 69 percent have white-collar jobs, 54 percent have less physically demanding jobs, 49 percent have less psychosocially demanding jobs, and 33 percent are manual workers. The coefficients of our 2SLS regressions in Fig. 7 are close to zero and are not statistically significant.

In addition, we investigate the effect of extra schooling on health behavior. Schooling could help people make healthier choices by teaching them how their actions affect their health [33]. The literature tends to find mixed effects, where in some domains education improves healthy behavior related to smoking, in others not [13]. For example, expanding upper secondary and college education reduces smoking [34, 35]. Brunello et al. [36] show that smoking, drinking, exercise, and BMI contribute to the education-health gradient in the context of compulsory schooling. Davies et al. [37] estimate a decrease in the probability of smoking and increases in the probability of drinking alcohol as well as exercising as a result of a reform of compulsory schooling in the UK. However, Kemptner et al. [19] find that the German compulsory schooling reform did not affect smoking behavior. Regarding other dimensions of health behavior, Fletcher and Frisvold [38] show that attending college increases the likelihood of using preventive care and [39] find no effect of the German compulsory schooling reforms on vaccination against COVID-19. We estimate the effect of the compulsory schooling reform on several dimensions of available (risky) health behaviors, namely present and past smoking habits, present alcohol consumption, the frequency of physical activity, and the adherence to a healthy diet. Our 2SLS coefficients shown in Fig. 7 are all not statistically significant and most are close to zero. The only exceptions are current smoking and alcohol consumption. The point estimate suggests an increase in the probability of currently smoking by about 6 percentage points and in drinking by 5 percentage points. However, both are not statistically distinguishable from zero.

Another possible explanation is that the compulsory schooling reform did not lead compliers to make better choices, such as less risky ones, which could have improved health. Therefore, we also estimate the effect of schooling on risk-taking behavior and locus of control, which is the perception of control over life outcomes as either internal or external. Existing literature finds mixed effects: Jung [40] estimates that a UK compulsory schooling reform increased self-reported risk-taking behavior, whereas [41] show how a Swedish compulsory schooling reform increased the likelihood of men to invest in the stock market (which is seen as riskier than, e.g., the bond market) and hold risky financial assets in general. Our results in Fig. 7 are not statistically significant and small.^9^ The German compulsory schooling reform did neither affect general risk-taking behavior nor the compliers’ perception of control over their lives.

In summary, while the compulsory schooling reform in Germany improved employment prospects, its effect on income is not crystal clear. The reform did not lead to healthier employment opportunities for compliers or to changes in alcohol or tobacco use, physical activity, or diet. It also did not affect risk-taking tendencies or locus of control. These factors could have contributed to better long-term health, which may explain why we do not observe lasting health effects from the reform.

## Conclusion

We study the relationship of education and health over the life-cycle using compulsory schooling reforms in West Germany as exogenous variation. Our main contribution to the literature is to estimate effects for different age groups starting age 30 and up to age 74, several decades after education took place. This allows to scrutinize a pattern that may have been missed in the previous literature: zero aggregate effects, as often found in the literature, might blur potential health effects that only show up late in life. Stronger effects in older ages can be justified theoretically [1] but may also be expected by descriptive results of an increasing education-health gradient over the life cycle, as found by previous studies, e.g., [3, 4].

While we find a slight increase in the health gap by education over the life cycle, we do not detect causal effects of an additional year of compulsory schooling on health and health care utilization for any age group. Most point estimates are practically zero with one possible exception: 2SLS estimates for self-rated health are statistically insignificant over the life cycle, but increase with age such that coefficient size for the oldest age groups may be economically relevant, albeit not statistically different from zero. Nevertheless, our simulated ex-post power analysis following [12] reveals that our sample does not suffer from power problems. We would be able to reliably detect true effects, even if they were less than half the size of these coefficients.

Of course, we only identify local average treatment effects, meaning effects for individuals who extended their education only because of the reform, that is, those at the lowest margin of willingness to continue schooling. The reform raised educational attainment for these compliers, improved their chances of being employed later in life, and has likely increased income (see [14]). However, it did not lead to healthier employment opportunities. The additional year of schooling also did not change health behaviors, risk preferences, or locus of control, all of which are possible channels through which education could affect health. This suggests that students might have already developed the skills needed to find healthier jobs and make healthier choices by the eighth grade, or that these skills are not influenced by the addition of a ninth grade, as [16] have previously argued.

This might be entirely different for other changes in the German educational system. For instance, the educational expansion in the 1960s to 1980s with a strong increase in the number of universities and high schools (*Gymnasien*) allowed many individuals to get much more education. Kamhöfer and Schmitz [15] do not only find positive (physical) health effects of this reform for individuals decades later but also that better jobs are a possible mechanism for this effect.

Germany has carried out several reforms of its education system in recent years, also for higher education margins such as university entrance diplomas. While these reforms – most notably the compression of secondary school education from 9 to 8 years, going along with increased instruction times – have been evaluated in terms of short-term health outcomes [42, 43], it cannot be ruled out that larger effects will only show up in some decades. Yet, as these reforms, again, most likely did not have significant effects on individuals’ career paths and chosen jobs, the results from this paper at least allow for the prediction that long-run health effects of these reforms might not be substantially larger than the short-term effects.

## Supplementary Information

Below is the link to the electronic supplementary material.

Supplementary file 1 (pdf 161 KB)

## Data Availability

The data that were used in this study are not publicly accessible and cannot be shared or published by the authors. However, they are available to any interested researcher upon individual registration with the German Socio-Economic Panel.

## Appendix A: Additional Figures and Tables

**Table 3:** Effect of education on health – previous economic literature

Authors	Year published	Country	Type	Instrument	Age group	Results
Adams [44]	2002	USA	Secondary	QOB	51 to 61	Positive effects
Arendt [45]	2005	Denmark	Middle school	CSR	25 to 64	No effects
Lleras-Muney [46]	2005	USA	Secondary	CSR	35 to 73	Reduction in mortality
Oreopoulos [47]	2006	UK	Secondary	CSR	32 to 64	Positive effects
Albouy and Lequien [48]	2009	France	Secondary	CSR	48 to 80	No effects
Silles [49]	2009	UK	Secondary	CSR	25 to 60	Positive effects
Braakmann [50]	2011	UK	Secondary	February birth	28 to 45	No effects
Kemptner et al. [19]	2011	Germany	Secondary	CSR	16 to 65	Women: no effects
						Men: positive effects
van Kippersluis et al. [51]	2011	Netherlands	Secondary	CSR	80 to 88	Reduction in mortality
Lager and Torssander [52]	2012	Sweden	Various	CSR	15 to 64	No effects
Clark and Royer [10]	2013	UK	Secondary	CSR	12 to 74	No effects
Jürges et al. [53]	2013	UK	Secondary	CSR	32 to 53,	No effects
					44 to 77	
Gathmann et al. [54]	2015	Europe	Secondary	CSR	50+	Women: no effects
						Men: positive effects
Palme and Simeonova [55]	2015	Sweden	Secondary	CSR	28 to 66	Negative effects
Brunello et al. [36]	2016	Europe	Secondary	CSR	50+	Positive effects
Buckles et al. [56]	2016	USA	College	War draft	28 to 65	Positive effects
Davies et al. [37]	2018	UK	Secondary	CSR	37 to 74	Positive effects
Meghir et al. [57]	2018	Sweden	Secondary	CSR	16 to 75	No effects
Kamhöfer et al. [15]	2019	Germany	College	College	39 to 68	Mental health: no effects
				expansion		Physical health positive e.
Dahmann and Schnitzlein [58]	2019	Germany	Secondary	CSR	50 to 85	No effects
Janke et al. [59]	2020	UK	Secondary	CSR	42 to 60	No effects (except diabetes)
Fischer et al. [60]	2021	Sweden	Secondary	CSR	18 to 81	Positive effects
Begerow and Jürges [22]	2022	Germany	Secondary	CSR	50 to 79	No effects
Malamud et al. [61]	2023	Romania	Secondary	CSR	42 to 71	No effects

Notes: Own research of studies without the claim of completeness. CSR = compulsory schooling reform, QOB = quarter of birth. The age ranges are not always clearly specified in the papers and sometimes deducted by ourselves using information provided on used birth cohorts as well as calendar years when the outcomes are measured. “No effects” usually means no significant effects and abstracts from economic effect sizes which might be non-zero. Brunello et al. [36] use various European countries

**Table 4:** SOEP versus external benchmarks

	Our sample, 2022	Full SOEP, 2022	External data benchmark	External data – year(s)	External data – source
Hospitalization	0.15	0.13	0.13	2008-2011	[62]
Poor or bad health	0.22	0.19	n.a.		
Number of diagnoses	1.69	1.52	n.a.		
BMI	27.07	26.63	26.00	2021	[63]
Age	69.00	52.43	44.70	2022	[64]
Female	0.50	0.52	0.51	2022	[65]
Employed	0.24	0.61	0.55	2023	[66]
Labor income	42,739	43,330	42,965	2021	[67]
Married	0.69	0.51	0.42	2022	[68]
Number of children	1.31	1.68	1.78	2024	[69]
Years of schooling	10.40	10.78	n.a.		
No degree	0.00	0.02	0.04	2021	[70]
Basic degree	0.35	0.24	0.29	2021	[70]
Intermediate degree	0.33	0.34	0.30	2021	[70]
High school degree	0.32	0.39	0.34	2021	[70]

Notes: The second column shows means over the entire main sample for the year 2022 with weights available in SOEP. The third column shows means from external benchmarks, the fourth which year(s) they are referring to and the last where they were obtained. The variables are defined as follows: Hospitalization is an indicator variable for whether a person was hospitalized for at least one night in the past year. Poor or bad self-rated health is a binary indicator of checking one of the two lowest of five possible categories of self-rated health. Number of diagnoses is a count variable of the total number of diagnosed (chronic) conditions and diseases from a list of 13 possible options. BMI is weight (in kilograms) divided by squared height (in meters). Employed is an indicator of whether a person is working at least part-time in a given survey year. Labor income is gross yearly income in Euros conditional on being employed at least part time. Details on data availability and variable construction: For subjective measures such as self-rated health, survey datasets like SOEP provide the most consistent large-scale sources. Furthermore, there are no population-based administrative datasets that record the number of diagnosed (chronic) conditions per individual. Measures based on health insurance claims data would—by design—exclude healthy, undiagnosed, or non-claiming individuals. Therefore, we do not include benchmarks for these two outcome variables. The mean number of diagnoses in SOEP refers to 2023, as this variable is collected only every two years. The external benchmark for the employment share is defined as the number of employed persons residing in Germany divided by the total German population. The external benchmark for the share married is the number of individuals who are married or in a registered partnership divided by the total German population. SOEP covers individuals aged 16 and older, while the benchmark includes the entire population, which directly affects differences in BMI, average age, employment share, and share married. The external benchmark for the number of children is calculated as the number of family members in households with opposite-sex spouses minus two. Basic degree refers to finishing *Hauptschule*. Intermediate degree refers to finishing *Realschule* or *polytechnische Oberschule*, its equivalent in the former German Democratic Republic (East Germany). High school degree refers to *(Fach-) Hochschulreife*

## Appendix B: Simulated ex-post power analysis

Detailed steps of the power analysis in “A simulated ex-post power analysis” section, leading to Fig. 4:

1. From the baseline sample, which includes individuals born within five years of the state-specific pivotal cohorts, eliminate all observations affected by the reform. This includes everyone born in the pivotal cohorts or later. Thus, we only use “untreated” observations to ensure that possible treatment effects do not affect our results. This process involves discarding 8,819 out of 15,913 observations containing information on the number of diagnoses, while retaining 7,094.
2. Refill the sample so that, for each federal state, the number of observations per age group is the same as before. First, we use data from individuals born between six and ten years before the pivotal cohort. For the remaining observations, we oversample individuals who were not treated.
3. Randomly select individuals from the sample created in Step 2 and assign them the treatment (*reform*), ensuring that the proportion of treated individuals in each federal state is the same as in the original sample.
4. Assign the treated individuals a uniform and constant synthetic treatment effect of *X*.
5. Estimate the reduced-form regression and check whether the estimated coefficients of the treatment effects in each age group is significant at the 5% level, i.e., whether or not we made a type-II-error (failing to reject the null hypothesis of a zero effect although we know that the true effect is *X ≠ 0*).
6. Repeat steps 3 to 5 1,000 times and count the share of significant treatment effect estimates for each level of *X*.
7. Repeat step 6 30 times while increasing the imposed treatment effect *X* is gradually increased from -0.001 to -0.03 in steps of 0.001.

Figure 4 reports the results of this exercise where for each of the 30 imposed treatment effects between 0.001 and 0.03 we run 1,000 reduced-form regressions, each time randomizing who receives treatment, and calculate the share of statistically significant coefficients. Figure 19 reports the results for a comparable approach with BMI as the outcome.

## Appendix C: Information on additional outcomes

Generally, all additional outcomes we are investigating in “Effects on non-health outcomes” section are available in SOEP or can be constructed from information in SOEP. Below, we provide additional details on these variables.

### Labor market outcomes

Employed is an indicator variable equal to one if a respondent is employed in a full or part time job in a given year of data collection, zero if not. Works part time is an indicator variable equal to one if a respondent is working a part-time job in a given year of data collection, zero if not. Unemployed is an indicator variable equal to one if a respondent is unemployed in a given year of data collection, zero if not. Income refers to the natural logarithm of individual labor earnings, which includes wages and salary from all employment including training, primary and secondary jobs, and self-employment, plus income from bonuses, overtime, and profit-sharing. Occupations are classified as physically (psychosocially) highly demanding if the Overall Physical (Psychosocial) Exposure Index for the occupation derived by [71] is larger than five and as less demanding if it is less or equal to five, as done in [72]. We group the occupations into manual and non-manual based on the 11 classes of the Erikson and Goldthorpe (EGP) class schema. In SOEP, the EGP is derived from the ISCO-88 classification as well as the information on self-employment and number of employees/supervisory status [73]. EGP classes I, II, III, IVa, IVb and V are classified as non-manual, and classes VI, VII and IVc as manual. See Table 5 for details. We restrict the sample to those within the working age group i.e. 30 – 65 years.

### Health behavior

Currently smoking is an indicator variable equal to one if and individual is smoking in a given year of data collection. Ever smoked is an indicator variable equal to one if an individual has mentioned they every smoked or if they indicated they were currently smoking in a previous year of data collection. Consumes alcohol is an indicator variable equal to one if an individual consumed alcohol in a given year of data collection. Sport >1 per week is an indicator variable equal to one if an individual does sports at least once per week. Sport <1 per month is an indicator variable equal to one if an individuals does sports less than once a month. Sport never is an indicator variable equal to one if an individual indicated they never do sports. Healthy diet: high adherence is an indicator variable equal to one if an individual adheres strongly or very strongly to a healthy diet. Healthy diet: low adherence is an indicator variable equal to one if an individual adheres a little or not at all to a healthy diet.

**Table 5:** Job classifications

EGP classification	Manual/Non-manual
(I) Higher Managerial and Professional Workers	Non-manual worker
(II) Lower Managerial and Professional Workers	Non-manual worker
(IIIa) Routine Clerical Work	Non-manual worker
(IIIb) Routine Service and Sales Work	Non-manual worker
(IVa) Small Self-Employed with Employees	Non-manual worker
(IVb) Small Self-Employed without Employees	Non-manual worker
(V) Manual Supervisors	Non-manual worker
(VI) Skilled Manual Workers	Manual worker
(VIIa) Semi- and Unskilled Manual Workers	Manual worker
(VIIb) Agricultural Labour	Manual worker
(IVc) Self-Employed Farmers	Manual worker

*Source:* SOEP Group [73]. *Notes:* Own grouping for manual/non-manual and calculation based on SOEP and NEPS

### Preferences and traits

Willingness to take risks is a scale for self-assessed general willingness to take risks ranging from 0 (not at all risk-taking) to 10 (very risk-taking). Locus of control is a factor variable summarizing eight dimensions of locus of control into one singular variable. It is standardized to have mean zero and standard deviation of one. Positive values refer to internal, negative values to external locus of control. For details and descriptions of how to construct this factor variable, see [74] and [75]. We adopted their best-practice approach in the construction of this variable.

## Footnotes

1 Regarding other outcomes, Bhuller et al. [8] and Delaney and Devereux [9] look at life-cycle effects of education on earnings.

2 The age groups begin at 35-39 for poor or bad health, 45-49 for BMI, and 55-59 for diagnoses because data for younger age groups is unavailable.

3 This entails that we have separate instruments by age group. For example, the relevant instrument for $Schoolyears \times age group_{50-54}$ is $Reform \times age group_{50-54}$.

4 Note that we are effectively using only one instrument, and that our regression specification with interactions by age group should essentially be understood as if we were running a separate regression for each age group with a single instrument. For this reason, we did not make adjustments for multiple instruments, but instead conducted a separate F-test for each age group.

5 The following section draws from the description of a similar approach in [29].

6 First stages are visualized in Fig. 9. The exact first-stage coefficients for the age groups 55–59, 60–64, 65–69, and 70–74 are 0.551, 0.587, 0.582, and 0.595, respectively.

7 Note that the SOEP is generally not the ideal data source for wage or other labor market analyses. The information is self-reported, and the sample size is smaller than that of more comprehensive administrative datasets, such as the Sample of Integrated Labor Market Biographies (SIAB).

8 For details on these or any other of the additional outcomes we are investigating here, please refer to Appendix C.

9 Note that all outcomes for which we show estimates in Fig. 7 are binary, except willingness to take risks (which is a scale from 0 to 10) and locus of control (which is a standardized factor variable). Therefore, confidence intervals on these variables seem a lot larger when displayed next to the confidence bands from the binary variables.

## Code Availability

The Stata code can be made available by the authors upon reasonable request.
