# Supplementary material for: Life-cycle health effects of compulsory schooling
Source: Eur J Health Econ. 2026 Jan 12;27(6):1293–322. doi: 10.1007/s10198-025-01884-2 (PMC13451446; doi:10.1007/s10198-025-01884-2)
Supplement: Supplementary file 1 [file 10198_2025_1884_MOESM1_ESM.pdf]

Supplementary Material

for

Life-cycle Health Effects of Compulsory Schooling

S1 Introduction

This supplement provides additions to the main paper by incorporating data from three extra sources: the German participants of the Survey of Health, Ageing and Retirement in Europe (SHARE), the National Educational Panel Study (NEPS), and the German Microcensus. It includes descriptions of these datasets, summary statistics, and baseline results estimated on the enlarged sample.

S2 Data and Descriptive Statistics

For this extended analysis, we pool data from four sources. The most comprehensive source is the German Socio-Economic Panel (SOEP), which serves as our baseline sample (see the main paper for a description).

We use SOEP version 40 containing yearly information from 1984 to 2023 [1]. We augment this baseline sample with observations from the Survey of Health Ageing, and Retirement, the German National Educational Panel Study and the German Microcensus. SHARE is a representative micro dataset which provides health and socio-economic information of people age 50 and older from 28 European countries and Israel. We consider waves 1, 2 and 4-9 [2, 3, 4, 5, 6, 7, 8, 9, 10]. Wave 3 (SHARELIFE) samples different individuals. NEPS is a longitudinal dataset that provides information on the acquisition of education in Germany, and educational processes and trajectories across the entire life span [11, 12]. We consider all 13 waves of the NEPS from 2007 to 2021. The German Microcensus (MZ) is a comprehensive, annual household survey conducted by the Federal Statistical Office of Germany. It provides detailed and representative data on the social, economic, and demographic structure of the population. Our final sample includes information from four waves of the Microcensus from 1999, 2003, 2009, and 2017 as these include information on height and weight, which we use to calculate BMI. The state an individual attended school in is only available in SOEP. We use the current state individuals live in as a proxy in the other data sets. See the Appendix of Begerow and Jürges [13] for an analysis that shows how in only 5% of the cases – in these data sets – this leads to a false assignment of the reform indicator which is used as an instrumental variable.

Table S1 reports numbers of observations in the final sample by outcome variable and age group. Next to the number of observations, we show from which data set the observations come. Clearly, SOEP has the most observations. Yet, as SHARE samples older individuals, it helps to increase numbers of observations particularly for the oldest age groups. Note that diagnoses and hospital visits are not included in the NEPS and Microcensus data. Microcensus is only used for weight-related outcomes and dominates the sample size there.

Table S1: Number of observations

Age group	Hospitalization last year: Obs.	Hospitalization last year: (% SOEP / %SHARE / % NEPS/ %MZ)	Poor or bad health: Obs.	Poor or bad health: (% SOEP / %SHARE / % NEPS/ %MZ)	Diagnoses: Obs.	Diagnoses: (% SOEP / %SHARE / % NEPS/) %MZ)	BMI: Obs.	BMI: (% SOEP / %SHARE / % NEPS/) %MZ)
30-34	4,316	(100 / 0 / 0 / 0)						
35-39	6,557	(100 / 0 / 0 / 0)	1,503	(100 / 0 / 0 / 0)				
40-44	7,203	(100 / 0 / 0 / 0)	5,140	(100 / 0 / 0 / 0)				
45-49	10,357	(100 / 0 / 0 / 0)	10,062	(100 / 0 / 0 / 0)			16,007	(14 / 0 / 0 / 86)
50-54	13,661	(95 / 5 / 0 / 0)	13,616	(94 / 5 / 1 / 0)	1,299	(47 / 53 / 0 / 0)	27,185	(18 / 3 / 0 / 79)
55-59	14,818	(90 / 10 / 0 / 0)	16,008	(84 / 9 / 8 / 0)	4,312	(67 / 33 / 0 / 0)	29,869	(21 / 5 / 1 / 74)
60-64	15,066	(86 / 14 / 0 / 0)	19,484	(67 / 11 / 22 / 0)	7,445	(72 / 28 / 0 / 0)	33,033	(19 / 6 / 2 / 73)
65-69	12,761	(85 / 15 / 0 / 0)	21,061	(52 / 9 / 39 / 0)	7,053	(72 / 28 / 0 / 0)	26,282	(20 / 7 / 7 / 66)
70-74	6,475	(85 / 15 / 0 / 0)	12,388	(44 / 8 / 48 / 0)	3,559	(72 / 28 / 0 / 0)	10,504	(24 / 9 / 14 / 52)

Notes: This table presents the number of observations by age group and the composition of our sample for all four health outcomes based on data from SOEP, NEPS, SHARE and MZ and our sample selection, as outlined in Chapter 2

The additional datasets are meant to supplement the SOEP. The Microcensus provides a large number of observations for which we have height and weight data (and thus BMI). The NEPS and SHARE datasets are intended to increase the number of observations for older age groups. In Table S2 we show the summary statistics from Table 2 for the combined sample as well as separately by dataset. Indeed, participants from NEPS and SHARE are older on average. NEPS participants have slightly more years of schooling and are more likely to be born after a compulsory schooling reform. The latter likely reflects heterogeneous sampling decisions across datasets. Compared to the SOEP benchmark, slightly more SHARE participants have been hospitalized. However, they have a slightly lower number of diagnosed chronic conditions, a somewhat higher BMI, and are significantly more likely to rate their health as poor or bad. NEPS participants are less likely to rate their own health as poor or bad.

Table S2: Summary statistics by dataset

	Combined sample		By dataset			
	Mean	SD	MZ	NEPS	SHARE	SOEP
Outcome variables						
Hospitalization	0.13	0.33	.	.	0.16	0.12
Number of diagnoses	1.72	1.61	.	.	1.64	1.75
Poor or bad health	0.18	0.39	.	0.08	0.32	0.20
BMI	26.42	4.49	26.30	26.70	26.96	26.74
Treatment and instrument						
Years of schooling	9.93	1.73	9.80	10.45	9.98	9.98
Reform	0.56	0.50	0.57	0.62	0.51	0.55
Other information						
Age	57.04	9.58	57.32	66.71	62.70	53.94
Female	0.50	0.50	0.50	0.48	0.54	0.51
Observations	221,531		110,066	19,776	7,187	84,531

Notes: This table presents means by dataset based on data from SOEP, NEPS, SHARE and MZ and our sample selection, as outlined in Chapter 2. The outcome variables are defined as follows: Hospitalization is an indicator variable for whether a person was hospitalized for at least one night in the past year. Poor or bad self-rated health is a binary indicator of checking one of the two lowest of five possible categories of self-rated health. Number of diagnoses is a count variable of the total number of diagnosed (chronic) conditions and diseases from a list of 13 possible options. BMI is weight (in kilograms) divided by squared height (in meters). Here, we calculate the number of observations as all observations for which there is nonmissing information for at least one outcome.

S3 2SLS Results

Figure S1 shows our main results from the second stage of the 2SLS estimations. The results are fully consistent with our SOEP-only main analysis. They show similar patterns and magnitudes of the coefficients. Estimates fluctuate around zero for all outcomes and age groups. For the number of diagnoses, we are able to present estimates from an additional age group (50-54) due to the larger sample.

Figure S1: Two-stage least squares results

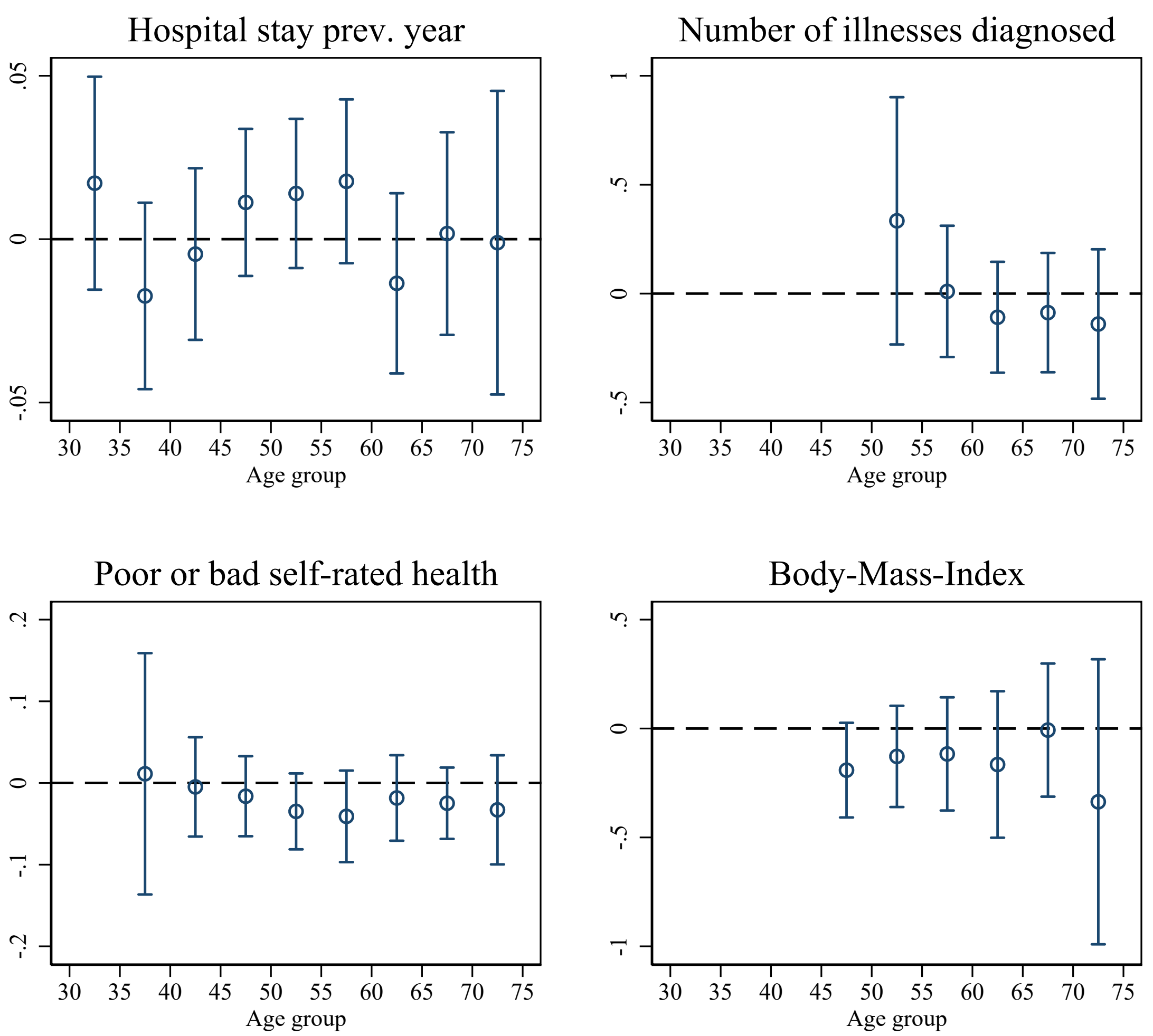

Notes: This figure shows estimates of the effect of years of schooling on four health outcomes across up to nine age groups. Hospital stay is an indicator variable for whether a person was hospitalized for at least one night in the past year. Number of diagnoses is a count of the total number of chronic conditions and diseases diagnosed from a list of thirteen possible options. Poor or bad self-rated health is an indicator variable for selecting one of the two lowest of the five possible self-rated health categories. BMI is weight (in kilograms) divided by height squared (in meters). Own calculations based on SOEP, SHARE, Microcensus, and NEPS. We visualize point estimates of coefficients β_g based on 2SLS Eq. (2) alongside 95% confidence intervals. Instruments are the interactions between the reform dummy variable (pivotal cohort and older) and the age groups. Coefficients plotted between 30 and 35 represent age group 30-34, and so on. Some age groups are missing due to data constraints. Standard errors clustered at state×year of birth level.

S4 Acknowledgements

This supplement uses data from SHARE Waves 1, 2, 4, 5, 6, 7, 8 and 9 (DOIs: 10.6103/SHARE.w1.900, 10.6103/SHARE.w2.900, 10.6103/SHARE.w4.900, 10.6103/SHARE.w5.900, 10.6103/SHARE.w6.900, 10.6103/SHARE.w7.900, 10.6103/SHARE.w8.100, 10.6103/SHARE.w8.900), see Börsch-Supan et al. [14] for methodological details. The SHARE data collection has been funded by the European Commission, DG RTD through FP5 (QLK6-CT-2001-00360), FP6 (SHARE-I3: RII-CT-2006-062193, COMPARE: CIT5-CT-2005-028857, SHARELIFE: CIT4-CT-2006-028812), FP7 (SHARE-PREP: GA N°211909, SHARE-LEAP: GA N°227822, SHARE M4: GA N°261982, DASISH: GA N°283646) and Horizon 2020 (SHARE-DEV3: GA N°676536, SHARE-COHESION: GA N°870628, SERISS: GA N°654221, SSHOC: GA N°823782) and by DG Employment, Social Affairs & Inclusion through VS 2015/0195, VS 2016/0135, VS 2018/0285, VS 2019/0332, and VS 2020/0313. Additional funding from the German Ministry of Education and Research, the Max Planck Society for the Advancement of Science, the U.S. National Institute on Aging (U01_AG09740-13S2, P01_AG005842, P01_AG08291, P30_AG12815, R21_AG025169, Y1-AG-4553-01, IAG_BSR06-11, OGHA_04-064, HHSN271201300071C, RAG052527A) and from various national funding sources is gratefully acknowledged (see www.share-project.org).

This supplement uses data from the National Educational Panel Study (NEPS; see Blossfeld and Roßbach [15]). The NEPS is carried out by the Leibniz Institute for Educational Trajectories (LIfBi, Germany) in cooperation with a nationwide network.

References

[1] SOEP. Socio-Economic Panel (SOEP), data for years 1984-2023, SOEP-Core v40, EU Edition, 2025.

[2] SHARE-ERIC. Survey of Health, Ageing and Retirement in Europe (SHARE) Wave 9. Release version: 9.0.0. SHARE-ERIC. Data set. DOI: 10.6103/SHARE.w1.900. Data set, 2024.

[3] SHARE-ERIC. Survey of Health, Ageing and Retirement in Europe (SHARE) Wave 9. Release version: 9.0.0. SHARE-ERIC. Data set. DOI: 10.6103/SHARE.w2.900. Data set, 2024.

[4] SHARE-ERIC. Survey of Health, Ageing and Retirement in Europe (SHARE) Wave 9. Release version: 9.0.0. SHARE-ERIC. Data set. DOI: 10.6103/SHARE.w4.900. Data set, 2024.

[5] SHARE-ERIC. Survey of Health, Ageing and Retirement in Europe (SHARE) Wave 9. Release version: 9.0.0. SHARE-ERIC. Data set. DOI: 10.6103/SHARE.w5.900. Data set, 2024.

[6] SHARE-ERIC. Survey of Health, Ageing and Retirement in Europe (SHARE) Wave 9. Release version: 9.0.0. SHARE-ERIC. Data set. DOI: 10.6103/SHARE.w6.900. Data set, 2024.

[7] SHARE-ERIC. Survey of Health, Ageing and Retirement in Europe (SHARE) Wave 9. Release version: 9.0.0. SHARE-ERIC. Data set. DOI: 10.6103/SHARE.w7.900. Data set, 2024.

[8] SHARE-ERIC. Survey of Health, Ageing and Retirement in Europe (SHARE) Wave 9. Release version: 9.0.0. SHARE-ERIC. Data set. DOI: 10.6103/SHARE.w8.900. Data set, 2024.

[9] SHARE-ERIC. Survey of Health, Ageing and Retirement in Europe (SHARE) Wave 9. Release version: 9.0.0. SHARE-ERIC. Data set. DOI: 10.6103/SHARE.w9.900. Data set, 2024.

[10] Axel Börsch-Supan, Martina Brandt, Christian Hunkler, Thorsten Kneip, Julie Ko rbmacher, Frederic Malter, Barbara Schaan, Stephanie Stuck, and on behalf of the SHARECentral Coordination Team Zuber, Sabrina. Data Resource Profile: The Survey of Health, Ageing and Retirement in Europe (SHARE). *International Journal of Epidemiology*, 42(4):992–1001, 06 2013. ISSN 0300-5771. doi: 10.1093/ije/dyt088.

[11] Hans-Peter Blossfeld, Jutta Von Maurice, and Thorsten Schneider. The Na tional Educational Panel Study: need, main features, and research potential. *Zeitschrift für Erziehungswissenschaft*, 14(2):5–17, 2011. doi: https://doi.org/10.1007/ s11618-011-0178-3.

[12] NEPS Network. National Educational Panel Study, Scientific Use File of Starting Cohort Adults., 2022.

[13] Tatjana Begerow and Hendrik Jürges. Does compulsory schooling affect health? Evidence from ambulatory claims data. *The European Journal of Health Economics*, 23 (6):953–968, 2022. doi: https://doi.org/10.1007/s10198-021-01404-y.

[14] Axel Borsch-Supan, Martina Brandt, Christian Hunkler, Thorsten Kneip, Julie Ko rbmacher, Frederic Malter, Barbara Schaan, Stephanie Stuck, and on behalf of the SHARECentral Coordination Team Zuber, Sabrina. Data Resource Profile: The Sur vey of Health, Ageing and Retirement in Europe (SHARE). *International Journal of Epidemiology*, 42(4):992–1001, 06 2013. ISSN 0300-5771. doi: 10.1093/ije/dyt088.

[15] Hans-Peter Blossfeld and Hans-Günther Roßbach, editors. *Education as a Lifelong Process: The National German Educational Panel Study (NEPS)*. Springer VS, edition zfe (2nd ed.) edition, 2019
